# Acute Effects of Polyphenols on Human Attentional Processes: A Systematic Review and Meta-Analysis

**DOI:** 10.3389/fnins.2021.678769

**Published:** 2021-05-24

**Authors:** Piril Hepsomali, Arno Greyling, Andrew Scholey, David Vauzour

**Affiliations:** ^1^Unilever R&D, Colworth Science Park, Bedford, United Kingdom; ^2^Unilever Foods Innovation Centre, Wageningen, Netherlands; ^3^Nutrition, Dietetics and Food, Monash University, Notting Hill, VIC, Australia; ^4^Department of Nutrition and Preventive Medicine, Norwich Medical School, University of East Anglia, Norwich, United Kingdom

**Keywords:** cognition, nutrition, simple RT, choice RT, vigilance, rapid visual information processing, brain, flavonoids

## Abstract

**Background:** The effects of polyphenols on cognitive functions have been extensively studied. Due to the large heterogeneity in the study designs, however, it is often difficult to interpret their efficacy. To address this issue, we conducted a systematic review and meta-analyses to examine whether acute polyphenol intake may have a beneficial effect on cognition and specifically on the accuracy and speed of attention.

**Methods:** PubMed and Scopus databases were systematically searched for studies published up to end of August 2020 following PRISMA guidelines (PROSPERO registration number: CRD42021232109). Only placebo-controlled human intervention trials that assessed acute effects of polyphenols on accuracy and speed of attention were included in the meta-analyses. When cognitive tasks were repeated over time, pooled means and standard deviations for intervention and placebo over repetitions separately for each task for both speed and accuracy were calculated. We also conducted separate analyses focusing only on the last repetition. Furthermore, confounding effects of age and source of polyphenols were also considered.

**Results:** Eighteen studies met the inclusion criteria. The pooled analysis of last task repetitions showed that the acute consumption of polyphenols improved rapid visual information processing speed in young participants (*SMD* = 0.26; 95%*CI* = [0.03–0.50]; *I*^2^= 0%; *p* = 0.02; *k* = 5). All other analyses did not reach significance.

**Conclusion:** The results of the current study indicate that acute polyphenol consumption might improve speed in rapid visual information processing task, a higher order task with elements of vigilance, working memory, and executive function, in young participants; however, as the current literature is inconsistent and limited, further acute intervention studies are warranted to achieve more conclusive results.

## Introduction

Polyphenols constitute a large group of bioactive phytochemicals in the plant kingdom. They are classified in terms of the number of phenol rings that they encompass, the structural elements that bind the rings to each other as well as the substituents linked to them (Abbas et al., 2017). The main classes include (i) phenolic acids, (ii) stilbenes, (iii) lignans, and (iv) flavonoids. Phenolic acids are found in many fruit and vegetables, specifically in berries; stilbenes in red wine and grapes; and lignans in whole bran cereals and flaxseeds (for more examples please see Gomez-Pinilla and Nguyen, 2012; Figueira et al., 2017; Fraga et al., 2019). The main sources of flavonoids are berries, cocoa, citrus, tea, wine, and soy products (for more examples please see Gomez-Pinilla and Nguyen, 2012; Figueira et al., 2017; Fraga et al., 2019). As such, polyphenols are abundant in Mediterranean style and plant-forward diets (Figueira et al., 2017).

Although it has not been established whether polyphenols can cross the blood–brain barrier (BBB) to directly affect central mechanisms underlying cognitive processes (Schaffer and Halliwell, 2012), other physiologically plausible candidate modes of action have been identified. These include (i) reducing neuroinflammation, (ii) modulating the gut microbiota structure and function, (iii) activating the endogenous antioxidant defence system, and (iv) affecting the cardiovascular system to improve neurovascular coupling (Angeloni et al., 2020; Lamport and Williams, 2020).

Regardless of their modes of action, it has been well-documented that prolonged consumption of certain phenolic compounds and polyphenol-rich foods, and adherence to polyphenol-rich plant-forward or plant-based diets may contribute to limiting age-related neurodegeneration and preventing or slowing cognitive decline in older adults (Letenneur et al., 2007; Figueira et al., 2017; Vauzour, 2017; Lefevre-Arbogast et al., 2018; Scarmeas et al., 2018; Solfrizzi et al., 2018; Rajaram et al., 2019; Shishtar et al., 2020). A recent meta-analysis of randomised controlled clinical trials focusing on chronic polyphenol administration, however, showed that only some polyphenols may have potential beneficial effects on specific domains of cognition and mainly in cognitively healthy older individuals. These included verbal learning/memory and visuospatial ability (Potì et al., 2019). On the other hand, the effects of acute polyphenol consumption on cognition have not been well-characterised (Bell et al., 2015). For instance, a number of studies have investigated the acute effects of polyphenol intake on measures of speed and accuracy of attention in recent years. Outcomes of these studies, however, are mixed. While some studies showed positive acute effects of polyphenols, particularly in tasks that measure speed of attention (Scholey et al., 2010; Field et al., 2011; Watson et al., 2015; Dietz et al., 2017), others failed to show such beneficial effects (Kennedy et al., 2010; Bondonno et al., 2014; Wightman et al., 2014). Furthermore, given the heterogeneity in study quality, design, and polyphenol type and dosage, it is challenging to reach a definite conclusion whether (i) acute polyphenol intake improves cognition when compared to a matching placebo/control, and (ii) whether this improvement is specific to a type of polyphenol, age group, and/or cognitive domain only. To address this knowledge gap, we performed a systematic review and multiple meta-analyses of controlled human studies focusing on the acute effects of polyphenol and/or polyphenol-rich food intake on speed and accuracy of attention.

## Method

The protocol for this systematic review and meta-analyses was registered in the international prospective register of systematic reviews (PROSPERO, registration number: CRD42021232109) and the articles were selected according to the Preferred Reporting Items for Systematic Reviews and Meta-Analyses (PRISMA) diagram (Moher et al., 2015; Shamseer et al., 2015).

### Data Sources and Search Strategy

We carried out electronic literature searches on PubMed and Scopus to identify relevant studies. The search was conducted until the end of August 2020. Search strategy could be found in Supplementary Material.

### Inclusion and Exclusion Criteria

One reviewer (PH) independently selected papers according to the following inclusion and exclusion criteria: **Inclusion Criteria;** (i) Treatment: polyphenols (no dose or type limit); (ii) Treatment duration: acute studies only (within a day); (iii) Outcome measures: accuracy and speed of attention measured by computerised tasks; (iv) Design: randomised controlled trials; (v) Participants: Any age or gender, healthy participants. **Exclusion Criteria**; (i) Design: Case report, letter to editor, conference paper, thesis, personal opinion, or commentary; (ii) Animal studies, *in vitro* and *ex vivo* studies, psychophysiological and/or neuroimaging studies.

### Selection of Studies

The outcome measures of attention shown below were included in the systematic review/meta- analysis. We only included attention measures that were used across eight or more individual studies and these were simple reaction time (RT), choice RT, digit vigilance (DV), and rapid visual information processing (RVIP) tasks. In the simple RT task, we only used speed of reaction (ms) data. In choice RT, digit vigilance and RVIP tasks, we used both accuracy (% correct) and speed (ms) data. Short descriptions of simple RT (Wightman et al., 2012; Bondonno et al., 2014; Massee et al., 2015; Dietz et al., 2017; Haskell-Ramsay et al., 2017, 2018a; Kennedy et al., 2017; Watson et al., 2019), choice RT (Field et al., 2011; Wightman et al., 2012; Bondonno et al., 2014; Massee et al., 2015; Dietz et al., 2017; Haskell-Ramsay et al., 2017, 2018a; Kennedy et al., 2017; Watson et al., 2019), digit vigilance (Bondonno et al., 2014; Watson et al., 2015, 2019; Keane et al., 2016; Dietz et al., 2017; Haskell-Ramsay et al., 2017, 2018a; Kennedy et al., 2017), and RVIP (Kennedy et al., 2010, 2020; Scholey et al., 2010; Cropley et al., 2012; Wightman et al., 2012, 2014, 2018; Massee et al., 2015; Watson et al., 2015; Keane et al., 2016; Haskell-Ramsay et al., 2018a; Philip et al., 2019) tasks are available elsewhere.

### Data Extraction

Two review authors (PH and AG) independently extracted data to evaluate and classify the quality of each study. Any disagreements were discussed between the authors until resolution. The following data was extracted by from all publications: (i) publication details: authors, year, journal; (ii) participant characteristics: number of participants recruited, number of participants included in the study, number of participants (intervention), number of participants (control), gender, and age range; (iii) study design: design and blinding; (iv) intervention characteristics: dose and type of polyphenols consumed; (v) control characteristics: presence/absence of control/placebo, control/placebo doses and types; (vi) outcome measures: accuracy and speed of attention measured by computerised tasks; (vii) remarks: notes on the factors that might affect results/data quality.

We emailed authors and asked them to provide data missing from included studies where necessary. In cases where the authors did not reply, we used calculations provided in Systematic Reviews of Interventions to obtain missing means and standard deviations (Higgins et al., 2020).

### Quality and Risk of Bias Assessment

One reviewer (PH) evaluated the quality of the studies included in this review by using the Cochrane Collaboration's tool for assessing risk of bias in randomised trials (Higgins et al., 2011). For this purpose, 7 different domains were considered (random sequence generation, allocation concealment, blinding of participants and personnel, blinding of outcome assessment, incomplete outcome data, selective reporting, and other sources of bias). Publication bias was evaluated by means of visual inspection of funnel plots (constructed by inverse plotting SE against the respective SMD for each measure of speed and accuracy of attention) and Egger's regression test (with *p* < 0.1 indicating asymmetry) (Egger et al., 1997). Heterogeneity was assessed by means of the Cochran's *Q* statistic (significant at *p* < 0.1) and quantified by the *I*^2^ statistic (with values of 25, 50, and 75% considered to be low-, moderate-, and high-level heterogeneity, respectively) (Higgins et al., 2003).

### Statistical Analysis

All statistical meta-analyses and graphical display were conducted using Jamovi 1.2.27 (The Jamovi Project, 2020) that utilises MAJOR meta-analysis module, which relies on *metafor* package for R (Hamilton, 2018). We selected highest polyphenol dose to include in the meta-analyses where studies had 2 or more intervention groups.

Separate random effects models were used to examine the effect of polyphenols on speed and accuracy of attention in simple RT, choice RT, digit vigilance, and RVIP tasks. All effect sizes were calculated using the standardised mean differences (SMD) due to the heterogeneity of cognitive tasks used. SMD effects sizes were interpreted using rules of thumb (<0.40 = small, 0.40–0.70 = moderate, >0.70 = large effect) (Higgins et al., 2020). We considered a statistically significant finding with *p* < 0.05.

## Results

### Summary of Included Studies

**Figure 1:**
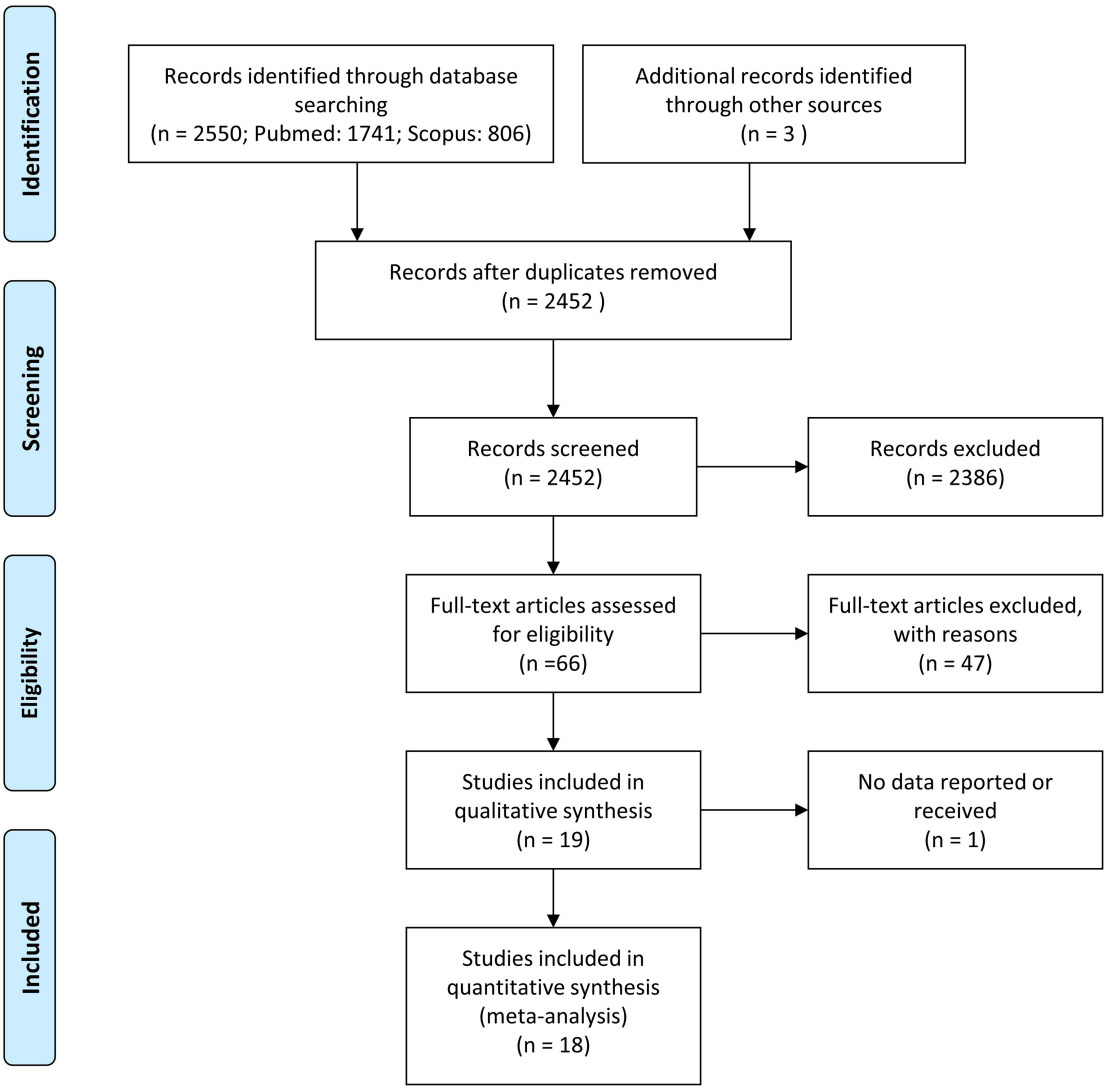
PRISMA flowchart of the selection procedure.

2,250 studies were identified using our defined search strategy. After screening for eligibility applying our inclusion and exclusion criteria described in the method section, 18 studies that met our criteria were included in the current review and meta-analyses (see Figure 1). Summaries of all the studies are presented in Table 1.

**Table 1:** Summary table of included studies.

**References**	**Participants**	Intervention (I) vs. Control (C)^*^	**Design**	**Cognitive tasks**	Results^**^
Bondonno et al. (2014)	30 (6 Males, Age Range: 33–61)	I: 80 g apple skin and 120 g apple flesh (184 mg of total quercetin glycosides and 180 mg of (–)-epicatechin) C: 120 g apple flesh (<5 mg of total quercetin glycosides and (–)-epicatechin)	Blinding unknown, controlled, crossover	Simple RT Choice RT Digit Vigilance	Simple RT – Speed: n.s. Choice RT – Accuracy: n.s.; Speed: n.s. Digit Vigilance – Accuracy: n.s.; Speed: n.s.; False Alarms: n.s.
Cropley et al. (2012)	39 (20 Males, Age Range: 53–79)	I: 6 g decaf coffee (521 mg total CGA and 11 mg caffeine) C: 6 g of placebo coffee (0 mg total CGA and 0 mg caffeine)	Double-blind, controlled, crossover	RVIP	Accuracy: n.s.; Speed: n.s.
Dietz et al. (2017)	23 (4 Males, Age Range: 20–35)	I: 4 g matcha powder in tea or bar (67 mg L-theanine, 280 mg EGCG, and 136 mg caffeine) C: 2.5 g spinach powder	Single-blind, controlled, crossover	Simple RT Choice RT Digit Vigilance	Simple RT – Speed: faster responses in the matcha tea (vs. control) condition Choice RT – Accuracy: n.s.; Speed: faster responses in the matcha (vs. control) condition Digit Vigilance – Accuracy: n.s.; Speed: n.s.; False Alarms: n.s.
Field et al. (2011)	30 (8 Males, Age Range: 18–25)	I: 35 g of the dark chocolate (773 mg of CF, 38 mg caffeine, and 222 mg theobromine) C:35 g white chocolate (trace amounts of CF, caffeine, and theobromine)	Single-blind, controlled, crossover	Choice RT	Choice RT – Accuracy: n.s.; Speed: faster responses in the intervention (vs. control) condition during the predictable (but not unpredictable) phase of the task
Haskell-Ramsay et al. (2017)	20 (7 Males, Age Range: 18–35)	I: 200 ml Welch's™ purple grape juice and 30ml Schweppes™ blackcurrant flavour cordial (Phenolic content: 1504.5 mcg/ml; 346 mg polyphenols;31.8 mg anthocyanins) C: 200 ml Welch's™ white grape juice, 10 ml blackcurrant flavour cordial, and 20 ml cold water (Phenolic content: 135.1 mcg/ml)	Single-blind, controlled, crossover	Simple RT Choice RT Digit Vigilance	Simple RT – Speed: not reported Choice RT – Accuracy and Speed: not reported Digit Vigilance – Accuracy, Speed, and False Alarms: not reported Composite attention measure – Accuracy: n.s.; Speed: faster responses in the intervention (vs. control) condition
Haskell-Ramsay et al. (2018a)	60 (27 Males, Age Range: 18–65)	I: 500 mg brown seaweed extract tablet (>20% chlorogenic acid equivalent) C: placebo tablet	Double-blind, controlled, parallel	Simple RT Choice RT Digit Vigilance	Simple RT – Speed: n.s. Choice RT – Accuracy: reduced accuracy at the 120- and 160-min assessments in the control group when compared to the first assessment post-lunch, which was not apparent in the intervention group; Speed: n.s. Digit Vigilance – Accuracy: increased accuracy in the intervention (vs. control) condition during repetition 1 of the assessments; Speed: n.s.; False Alarms: n.s.
Haskell-Ramsay et al. (2018b)	59 (30 Males, Age Range: 22–74)	I: 220 ml regular coffee (100 mg caffeine) C: 220 ml water (2.5 g coffee flavouring)	Double-blind, controlled, crossover	Simple RT Digit Vigilance RVIP	Simple RT – Speed: n.s. Digit Vigilance – Accuracy: n.s; Speed: faster responses in intervention (vs. placebo) condition; False Alarms: n.s. RVIP – Accuracy: n.s; Speed: faster responses in intervention (vs. placebo) condition; False Alarms: n.s.
Keane et al. (2016)	27 (Unknown, Age Range: 45–60)	I: 60 ml Montmorency tart cherry concentrate (68.0 mg cyanidin-3-glucoside/l, 160.75 mean gallic acid equivalent/l and 0.59 mean Trolox equivalent/l) C: fruit flavoured cordial mixed with water, whey protein isolate, and maltodextrin (8·26 mean gallic acid equivalent/l)	Double-blind, controlled, crossover	Digit Vigilance RVIP	Digit Vigilance – Accuracy: n.s; Speed: n.s. RVIP – Accuracy: n.s; Speed: n.s.
Kennedy et al. (2010)	22 (Unknown, Age Range: 18–25)	I: 500 mg trans-resveratrol C: inert placebo capsule	Double-blind, controlled, crossover	RVIP	RVIP – Accuracy: n.s.; Speed: n.s.; False Alarms: n.s.
Kennedy et al. (2017)	59 (19 Males, Age Range: 40–65)	I: 1,600 mg of green oat extract [flavonoid content, calculated as isovitexin, of ≥0.3% (w/w)] C: placebo capsule (maltodextrin)	Double-blind, controlled, crossover	Simple RT Choice RT Digit Vigilance	Simple RT – Speed: n.s. Choice RT – Accuracy: n.s.; Speed: n.s. Digit Vigilance – Accuracy: n.s.; Speed: n.s.; False Alarms: n.s.
Kennedy et al. (2020)	132 (40 Males, Age Range: 35–65)	I: 1,290 mg Cognitaven® - 900 mg green oat extract (phenolic content unknown) C: placebo capsule (maltodextrin)	Double-blind, controlled, parallel	RVIP	RVIP – Accuracy: n.s; Speed: n.s.
Massee et al. (2015)	40 (20 Males, Age Range: 18–40)	I: 3,058 mg T. cacao seed extract (250 mg catechin polyphenols and 5.56 mg caffeine) C: placebo tablet (inert cellulose powder)	Double-blind, controlled, parallel	Simple RT Choice RT RVIP	Simple RT – Speed: n.s. Choice RT – Accuracy: n.s.; Speed: n.s. RVIP – Accuracy: n.s.; Speed: n.s.
Philip et al. (2019)	30 (14 Males, Age Range: 18–25)	I: 600 mg grape (*Vitis vinifera L.)* and wild blueberry (*Vaccinium angustifolium*) (Memophenol™, Activ'Inside, Beychac et Caillau, France) extract (260 mg flavonoids) C: placebo capsule (maltodextrin)	Double-blind, controlled, crossover	RVIP	RVIP – Accuracy: a trend towards increased accuracy in intervention (vs. control) condition; Speed: n.s.; False Alarms: n.s.
Scholey et al. (2010)	30 (13 Males, Age Range: 18–35)	I: 994 mg CF C: 46 mg CF	Double-blind, controlled, crossover	RVIP	RVIP – Accuracy: n.s.; Speed: faster responses in the intervention (vs. control) condition at 30 and 40-min; False Alarms: n.s.
Watson et al. (2015)	36 (13 Males, Age Range: 18–35)	I: DelCyan™ Blackcurrant extract or 142 ml “Blackadder” blackcurrant fruit juice (525 ± 5 mg of polyphenols per 60 kg of bodyweight; 571 mg anthocyanins; 590 mg polyphenols and 552 mg anthocyanins; 599 mg polyphenols, respectively) C:control drink (0 mg polyphenols)	Double-blind, controlled, crossover	Digit Vigilance RVIP	Digit Vigilance – Accuracy: n.s; Speed: faster responses in treatment (vs. control) condition at repetition 1, 4, and 7 for “Blackadder” (but not DelCyan™) RVIP – Accuracy: n.s; Speed: attenuation in the reduction of accuracy in DelCyan™, but not “Blackadder,” (vs. control) condition, irrespective of repetition; False Alarms: n.s.
Watson et al. (2019)	9 (3 Males, Age Range: Unknown)	I: Blackcurrant juice (515.7 mg polyphenols; 118.7 mg anthocyanins) C: control drink, sugar, vitamin C, and flavour matched	Double-blind, controlled, crossover	Simple RT Choice RT Digit Vigilance	Simple RT – Speed: n.s. Choice RT – Accuracy: n.s.; Speed: slower responses in intervention (vs. control) condition Digit Vigilance – Accuracy: n.s.; Speed: n.s.; False Alarms: n.s.
Wightman et al. (2014)	23 (4 Males, Age Range: 19–34)	I: 250 mg trans-resveratrol capsule C: inert placebo capsule	Double-blind, controlled, crossover	RVIP	RVIP – Accuracy: n.s
Wightman et al. (2018)	140 (46 Males, Age Range:50–70)	I: 950 mg *Sideritis scardica* extract (58 mg polyphenols) C: placebo capsule (maltodextrin)	Double-blind, controlled, parallel	Choice RT RVIP	Choice RT – Accuracy: n.s.; Speed: n.s. RVIP – Accuracy: n.s; Speed: n.s.; False Alarms: reduced false alarms in intervention (vs. control) condition at repetition 4

* *Only highest dose polyphenol interventions were reported;*

** *Only intervention vs. control differences were reported. Composite attention measure results were reported only when individual test results were not reported by the authors; RT, reaction time; n.s., not significant; CGA, chlorogenic acids; RVIP, rapid visual information processing; EGCG, epigallocatechin gallate; CF, cocoa flavanols; w/w, weight for weigh*.

### Meta-Analyses on Speed and Accuracy of Attention

**Figure 2:**
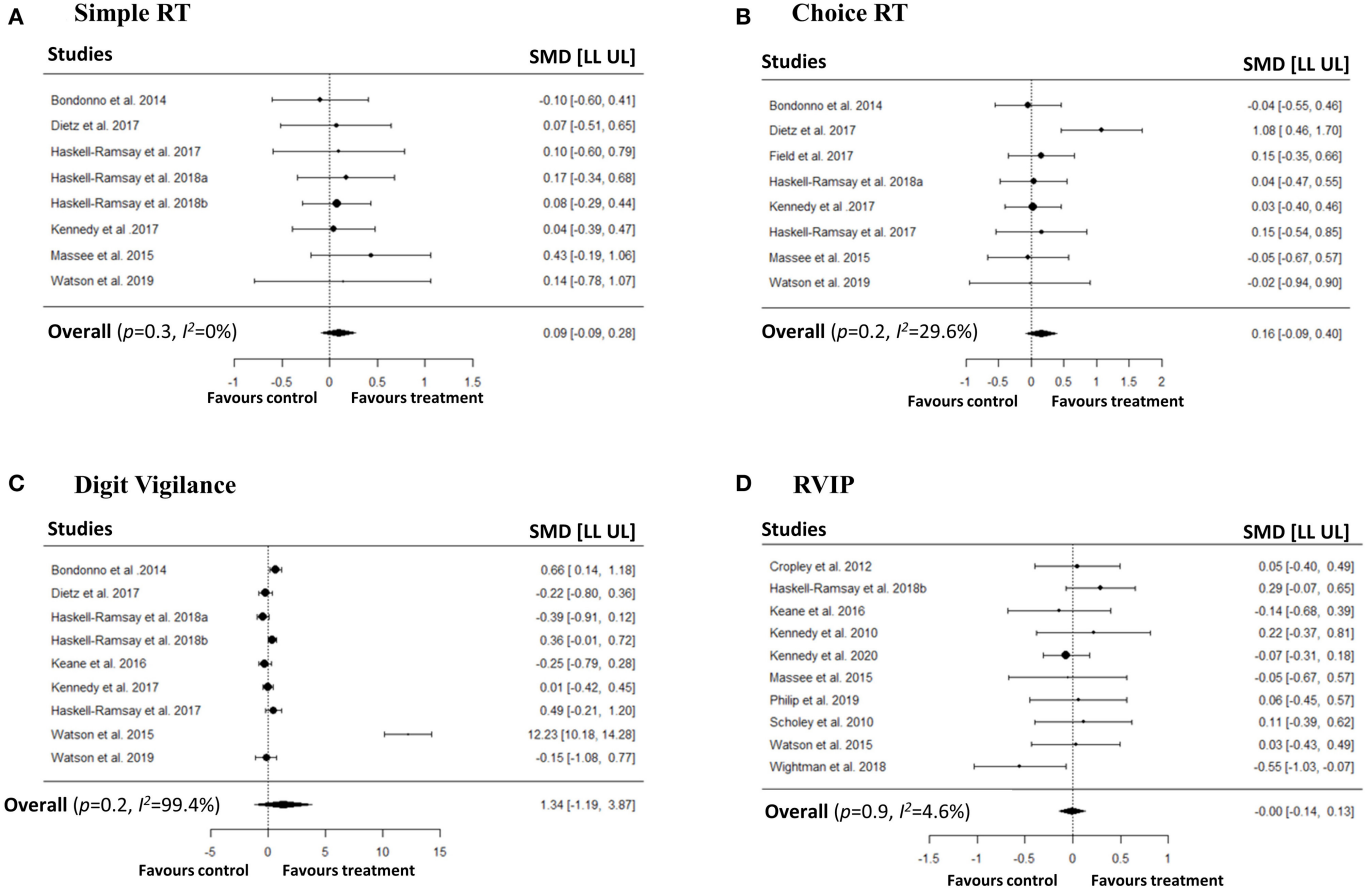
Meta-analyses on the effects of polyphenols on speed of attention (repetitions averaged) in **(A)** Simple RT, **(B)** Choice RT, **(C)** Digit Vigilance, **(D)** RVIP tasks.

**Figure 3:**
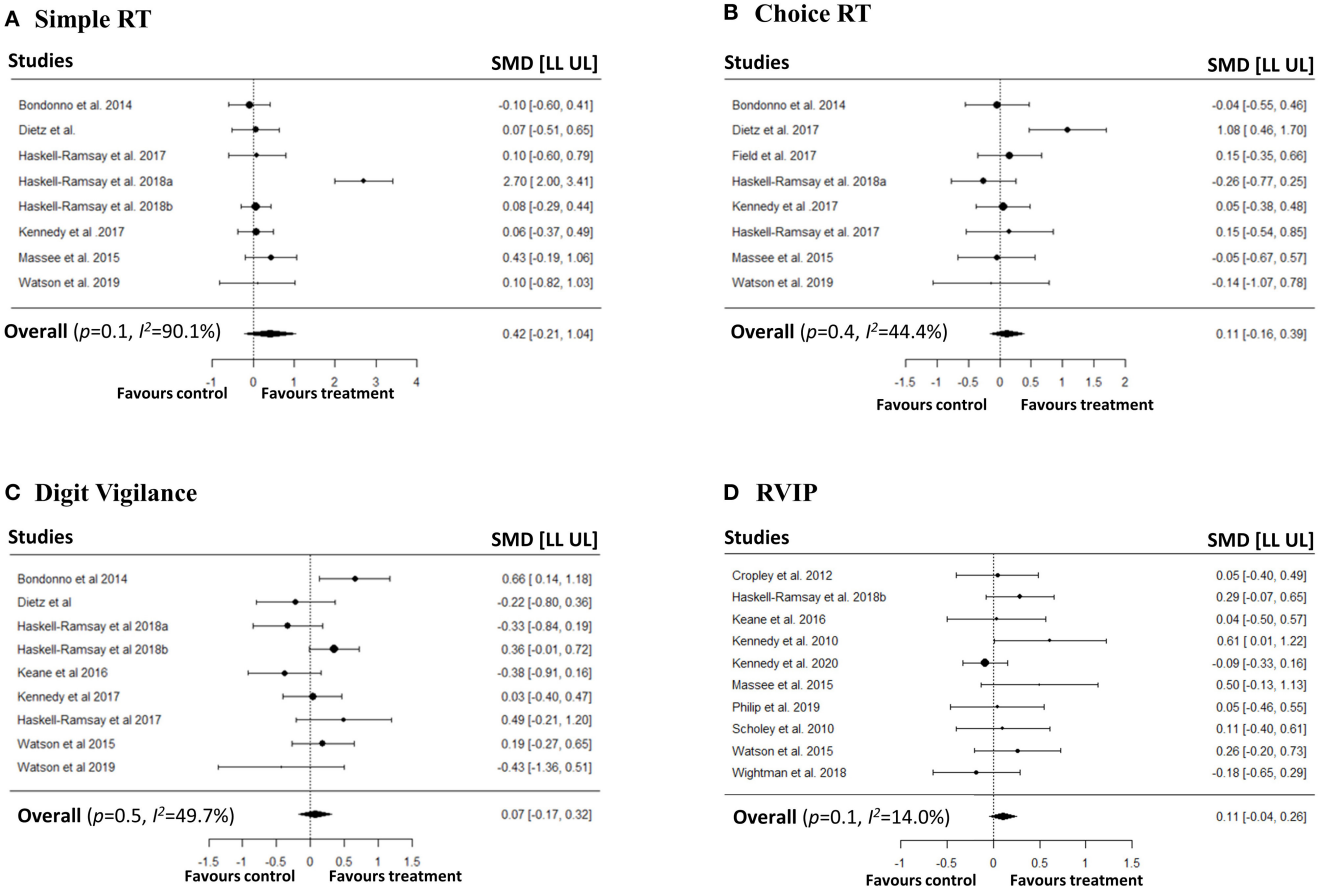
Meta-analyses on the effects of polyphenols on speed of attention (last repetition only) in **(A)** Simple RT, **(B)** Choice RT, **(C)** Digit Vigilance, **(D)** RVIP tasks.

**Figure 4:**
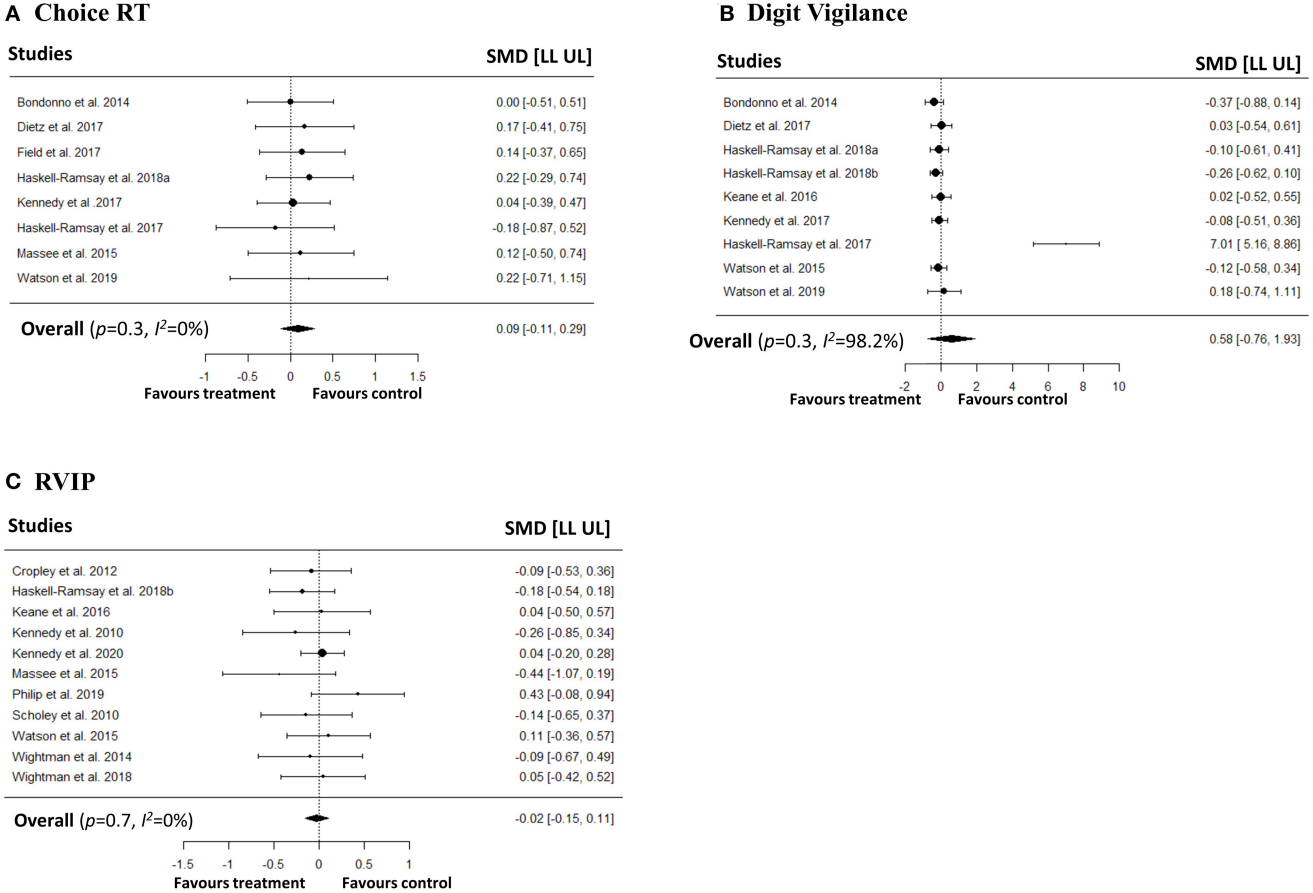
Meta-analyses on the effects of polyphenols on accuracy of attention (repetitions averaged) in **(A)** Choice RT, **(B)** Digit Vigilance, **(C)** RVIP tasks.

**Figure 5:**
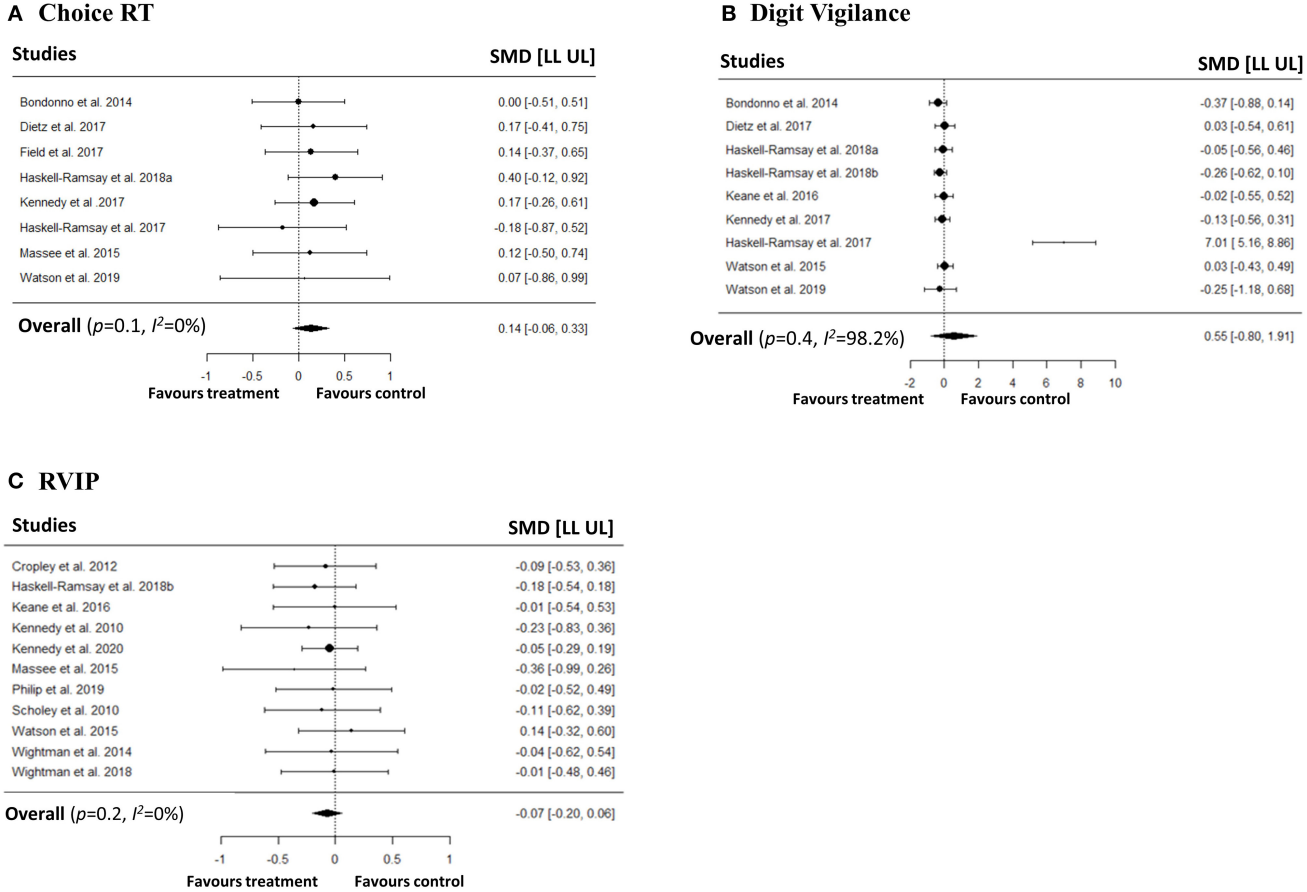
Meta-analyses on the effects of polyphenols on accuracy of attention (last repetition only) in **(A)** Choice RT, **(B)** Digit Vigilance, **(C)** RVIP tasks.

We conducted a series of meta-analyses with the aim of investigating the impact of polyphenols on both speed and accuracy of attention. In order to capture different attentional outcomes due to the disparate pharmacokinetic profiles of the polyphenols, we investigated both averaged repetitions and last repetition when cognitive tasks were repeated measures. No significant effects were observed on speed of attention in simple RT, choice RT, digit vigilance, and RVIP tasks in both averaged (Figure 2) and last repetitions (Figure 3). Similarly, no effects of polyphenol intake on accuracy of attention in choice RT, digit vigilance, and RVIP tasks in both averaged (Figure 4) and last repetitions (Figure 5) were observed.

### Sub-group Analyses

#### Polyphenol Source

There was only sufficient data to conduct sub-group analyses of studies on berry polyphenols. No statistically significant effects were found for any measures of speed- or accuracy of attention in this sub-group analysis (see Supplementary Material Results sections 1 and 3).

#### Age Groups

**Figure 6:**
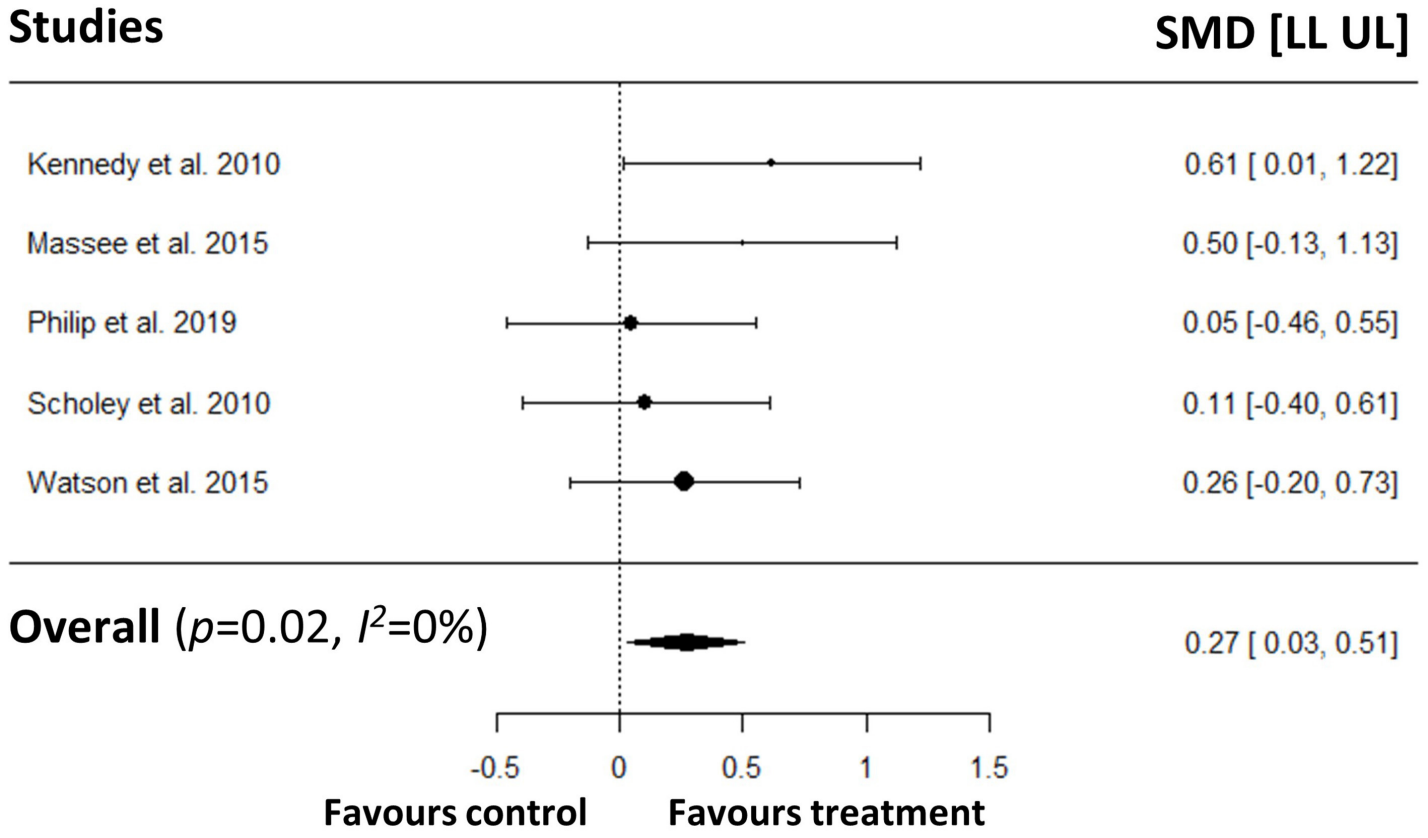
Meta-analysis on the effects of polyphenols on RVIP speed (last repetition only) in young participants.

In the averaged repetition analyses, no statistically significant effects on measures of speed of attention was seen in either of the age groups (see Supplementary Material Results sections 2.1 and 2.3). In the last repetition analyses, however, we found that young participants had significantly faster responses in polyphenol condition only in the RVIP task (SMD = 0.26; 95%CI = [0.03–0.50]) (Figure 6). All other last repetition analyses revealed non-significant results (see Supplementary Material Results sections 4.1 and 4.3). For measures of accuracy of attention, no statistically significant effects were found in either of the age groups (see Supplementary Material Results sections 2.2, 2.4, 4.2, and 4.4).

### Risk of Bias and Heterogeneity

Nearly 70% of the studies were categorised as having low risk of selection (random sequence generation and allocation concealment), performance, attrition, reporting, and other sources of biases. Risk for detection bias was unclear as most of the studies did not report whether outcome assessment and statistical analyses were blinded or not (see Supplementary Figure 1).

#### Averaged Repetitions

For speed of attention, heterogeneity for simple RT was low (*p*-value for *Q* statistic = 0.96, *I*^2^= 0%), choice RT was low-moderate (*p*-value for *Q* statistic = 0.17, *I*^2^ = 29.64%), digit vigilance (*p*-value for *Q* statistic < 0.001, *I*^2^ = 99.45%) was high, and RVIP was low (*p*-value for *Q* statistic = 0.43, *I*^2^= 4.6%). For accuracy of attention, heterogeneity for choice RT was low (*p*-value for *Q* statistic = 0.99, *I*^2^= 0%), digit vigilance (*p*-value for *Q* statistic < 0.001, *I*^2^= 98.24%) was high, and RVIP was low (*p*-value for *Q* statistic = 0.76, *I*^2^= 0%).

Egger's linear regression test did not indicate potential presence of publication bias for speed of attention in simple RT (*p* = 0.65), choice RT (*p* = 0.70), and RVIP (*p* = 0.96) tasks. Similarly, no bias was detected for choice RT (*p* = 0.97), and RVIP (*p* = 0.53) task accuracies. However, we observed potential publication biases for digit vigilance speed (*p* = 0.001) and accuracy (*p* = 0.001) studies. Additionally, visual inspection of contour-enhanced funnel plots did not confirm an obvious presence of publication bias in majority of the studies with an exception for digit vigilance speed and accuracy (see Supplementary Figures 2, 4).

#### Last Repetition

For speed of attention, heterogeneity for simple RT was high (*p*-value for *Q* statistic < 0.001, *I*^2^= 90.1%), choice RT (*p*-value for *Q* statistic = 0.08, *I*^2^= 44.4%) and digit vigilance (*p*-value for *Q* statistic = 0.04, *I*^2^= 49.7%) was moderate, and RVIP was low (*p*-value for *Q* statistic = 0.38, *I*^2^= 14.01%). For accuracy of attention, heterogeneity for choice RT was low (*p*-value for *Q* statistic = 0.95, *I*^2^= 0%), digit vigilance (*p*-value for *Q* statistic < 0.001, *I*^2^= 98.2%) was high, and RVIP was low (*p*-value for *Q* statistic = 0.99, *I*^2^ = 0%). Heterogeneity for speed in the RVIP task in young participants was also low (*p*-value for *Q* statistic = 0.57, *I*^2^ = 0%).

Egger's linear regression test did not indicate potential presence of publication bias for speed of attention in simple RT (*p* = 0.44), choice RT (*p* = 0.82), digit vigilance (*p* = 0.38), and RVIP (*p* = 0.07) tasks. Similarly, no bias detected for choice RT (*p* = 0.57), and RVIP (*p* = 0.76) task accuracies. Also, no bias detected for RVIP speed studies in young participants (*p* = 0.20). However, we observed a potential publication bias for digit vigilance accuracy (*p* = 0.001) studies. Additionally, visual inspection of contour-enhanced funnel plots did not confirm an obvious presence of publication bias in majority of the studies with an exception for digit vigilance accuracy (see Supplementary Figures 3, 5).

## Discussion

The importance of intact attentional performance in young adults and the need of maintaining attention in older adults have been well-established. This area, however, has not previously been systematically examined in relation to acute polyphenol intake. Hence, the aim of the current review and meta-analyses was to investigate the acute effects of polyphenol and/or polyphenol-rich food intake on cognitive performance, with a particular focus on speed and accuracy of attention. In this meta-analysis, summarising evidence from 18 randomised controlled intervention studies, we observed faster RVIP responses only in the subset of younger participants following acute consumption of polyphenols.

One might argue that our finding in relation to the beneficial effect of polyphenols on RVIP speed in younger participants may be attributable to caffeine's cognitive-enhancing effect (Nehlig, 2010). However, in the current meta-analyses, only two studies out of five that utilised RVIP task in young participants used small amounts of caffeine in their treatment conditions (Scholey et al., 2010; Massee et al., 2015). Therefore, it is more likely that the effect of polyphenol-rich foods on RVIP speed was driven mainly by their polyphenol content. Consistent with our results, recent meta-analyses examining the differential effects of moderator variables in relation to participant characteristics and supplementation protocols have revealed beneficial effects of polyphenols in younger (vs. older) individuals and following acute (vs. chronic) supplementation (Ammar et al., 2020a,b). As it has been shown that even healthy older adults have relatively poor attention due to cognitive ageing (Filley and Cullum, 1994; Harada et al., 2013), acute polyphenol supplementation might not be helpful to delay or reverse these effects. On the other hand, as young adults have relatively intact attention, maintaining these processes with acute polyphenol supplementation might be a more realistic goal. Although supporting evidence comes from a recent theory that identifies young adults as candidate targets for antiaging interventions due to the possibility of preventing diseases in young (Belsky et al., 2015), further high-quality research is required to examine whether starting polyphenol interventions in young adults to delay and/or prevent cognitive decline would be more advantageous or not.

It is important to note that, for this systematic review and meta-analysis, we restricted our analysis to acute effects on attentional measures. However, previous research suggests that specific cognitive processes may be differentially affected by various polyphenols (Potì et al., 2019). For instance, Kesse-Guyot et al. (2012) observed a positive association between total polyphenol intake and performance in tasks that involved verbal/language skills but not with executive function. Similarly, a recent longitudinal study showed positive associations with (i) higher flavanol and flavan-3-ol intakes and global function and verbal/visual memory (ii) higher total flavonoid and flavonoid polymer intakes and visual memory, and (iii) higher flavanol intake and verbal learning (Shishtar et al., 2020). Hence, a thorough exploration as to which cognitive domains could be improved by polyphenol intake is warranted.

Apart from faster RVIP responses, no statistically significant effects were found in the other meta-analyses of digit vigilance, choice- and simple reaction time tasks. These outcomes could, in part, be explained by potential limitations of the current review: Firstly, the included studies featured a range of different polyphenols, doses and delivery formats. In this regard, differences in polyphenol bioavailability, metabolism, and structure could have had an influence on the outcomes of the meta-analyses. Indeed, different types of polyphenols vary in their pharmacokinetic profiles (Del Rio et al., 2010; Clifford et al., 2013), which may also be affected by the food and/or format in which they are consumed, as well as interindividual differences in bioavailability at the participant level (Gibney et al., 2019). In addition to bioavailability, a host of other factors (e.g., health status, genetic variance, sex, body mass index, age) have been identified that may impact interindividual variability in physiological responses to polyphenols (Cassidy and Minihane, 2017; Gibney et al., 2019; Landberg et al., 2019). Combined, heterogeneity in these factors within and between the studies included in the meta-analyses, may have inflated interindividual variation in responses, and thus limited the statistical power of studies and meta-analyses to detect potential effects. We attempted to address this limitation through sub-group analyses. However, due to lack of available data, sub-group analyses were limited to studies providing berries as a polyphenol source and, we were unable to investigate potential differences in efficacy of the different polyphenol classes. This is an important limitation since differences in efficacy between different types of polyphenol have been reported (albeit in chronic supplementation studies) (Kesse-Guyot et al., 2012; Potì et al., 2019; Shishtar et al., 2020). We were also not able to conduct subgroup or meta-regression analyses to investigate potential effects of dose. The available data is not consistent at the moment, as dose-dependent effects have been suggested for some polyphenol sources (Kennedy et al., 2000; Bell et al., 2015), but not others (Scholey et al., 2010). Further investigation across polyphenol classes and doses in studies specifically designed for this purpose to provide answers in this regard. We were also not able to correct for potential confounding effects of variations in background polyphenol intake between the studies. It has been suggested that individuals most likely to participate in nutrition intervention trials, are usually health-conscious with good baseline nutritional status (Young et al., 2020). This “self-selection bias” may limit the ability to measure a potential benefit on functional status. As such, healthy diets generally have a high polyphenol content and, none of the included studies evaluated polyphenol intake status. Also, although some trials (Scholey et al., 2010; Field et al., 2011; Cropley et al., 2012; Watson et al., 2015, 2019; Keane et al., 2016; Philip et al., 2019) included a low-polyphenol run-in period, it is unclear whether this would have been sufficient to control for any potential effects of background polyphenol intake. Furthermore, the bioavailability of gut-derived polyphenol metabolites is several-fold higher than that of their parent compounds (Van Duynhoven et al., 2014). These metabolites have been implicated in the beneficial effects of polyphenol interventions (Espin et al., 2017), but would not reach the circulation within the measurement timeframe of the acute studies included in this review. In this regard, longer-term studies aimed at determining the role that polyphenol metabolites play in their reported cognitive effects would be useful and future studies are warranted.

In conclusion, the results of the current review indicate that acute polyphenol consumption might improve speed in RVIP task in young participants, however, as the current literature is inconsistent and limited, further studies are warranted to (i) replicate our current findings showing faster RVIP responses in young participants (and lack of this effect in old participants) following acute polyphenol consumption, preferably by using different polyphenol sources and doses, while controlling for background polyphenol intake and/or diet quality, and (ii) examine whether these acute improvements might translate into sustained cognitive benefits (i.e., slower age-related decline) in longitudinal settings.

## Data Availability Statement

The original contributions presented in the study are included in the article/Supplementary Material, further inquiries can be directed to the corresponding authors.

## Author Contributions

PH and AG wrote the manuscript with input from AS and DV who also contributed to the revision of the manuscript critically for important intellectual content. All authors approved the submitted version.

## Conflict of Interest

AG is employed by Unilever. PH was employed by Unilever, however, she is currently affiliated with University of Roehampton. AS and DV have received research funding, consultancy, travel support, and speaking honoraria from various industrial companies.
