# Supplementary material for: Acute Effects of Polyphenols on Human Attentional Processes: A Systematic Review and Meta-Analysis
Source: Front Neurosci. 2021 May 24;15:678769. doi: 10.3389/fnins.2021.678769 (PMC8180591; doi:10.3389/fnins.2021.678769)
Supplement: Supplementary file 1 [file Table_1.DOCX]

***Supplementary File***

**1. Search Terms**

**1.1. Pubmed**

((((((((((((((((((((((cogni*[Title/Abstract]) OR (neurocogni*[Title/Abstract])) OR ("cognitive function*"[Title/Abstract])) OR ("cognitive performance*"[Title/Abstract])) AND (polyphenol*[Title/Abstract])) OR (flavon*[Title/Abstract])) OR (flava*[Title/Abstract])) OR (isoflavone*[Title/Abstract])) OR (proanthocyanidin*[Title/Abstract])) OR (anthocyanidin*[Title/Abstract])) OR ("epigallocatechin gallate*"[Title/Abstract])) OR (gallocatechin*[Title/Abstract])) OR ("epicatechin gallate*"[Title/Abstract])) OR (epigallocatechin*[Title/Abstract])) OR (epicatechin*[Title/Abstract])) OR (catechin*[Title/Abstract])) OR (theaflavin*[Title/Abstract])) OR (tannin*[Title/Abstract])) OR (thearubigin*[Title/Abstract])) OR (phenolic*[Title/Abstract])) OR ("phenolic acid*"[Title/Abstract])) OR (stilbene*[Title/Abstract])) OR (lignan*[Title/Abstract]).

**1.2. Scopus**

( TITLE-ABS-KEY ( cognit* ) OR TITLE-ABS-KEY ( neurocogni* ) OR TITLE-ABS-KEY ( "cognitive function*" ) OR TITLE-ABS-KEY ( "cognitive performance*" ) AND TITLE-ABS-KEY ( polyphenol* ) OR TITLE-ABS-KEY ( flavon* ) OR TITLE-ABS-KEY ( flava* ) OR TITLE-ABS-KEY ( isoflavone* ) OR TITLE-ABS-KEY ( proanthocyanidin* ) OR TITLE-ABS-KEY ( anthocyanidin* ) OR TITLE-ABS-KEY ( "epigallocatechin gallate*" ) OR TITLE-ABS-KEY ( gallocatechin* ) OR TITLE-ABS-KEY ( "epicatechin gallate*" ) OR TITLE-ABS-KEY ( epigallocatechin* ) OR TITLE-ABS-KEY ( epicatechin* ) OR TITLE-ABS-KEY ( catechin* ) OR TITLE-ABS-KEY ( theaflavin* ) OR TITLE-ABS-KEY ( tannin* ) OR TITLE-ABS-KEY ( thearubigin* ) OR TITLE-ABS-KEY ( phenolic* ) OR TITLE-ABS-KEY ( "phenolic acid*" ) OR TITLE-ABS-KEY ( stilbene* ) OR TITLE-ABS-KEY ( lignan* ) ).

**2. Risk of Bias**

**Supplementary Figure 1.** Risk of bias across studies.

**3. Funnel Plots**

| **A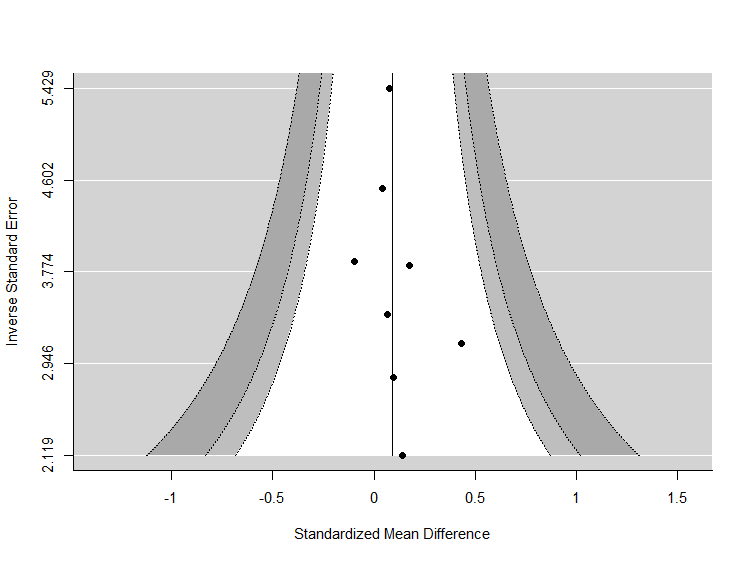** | **B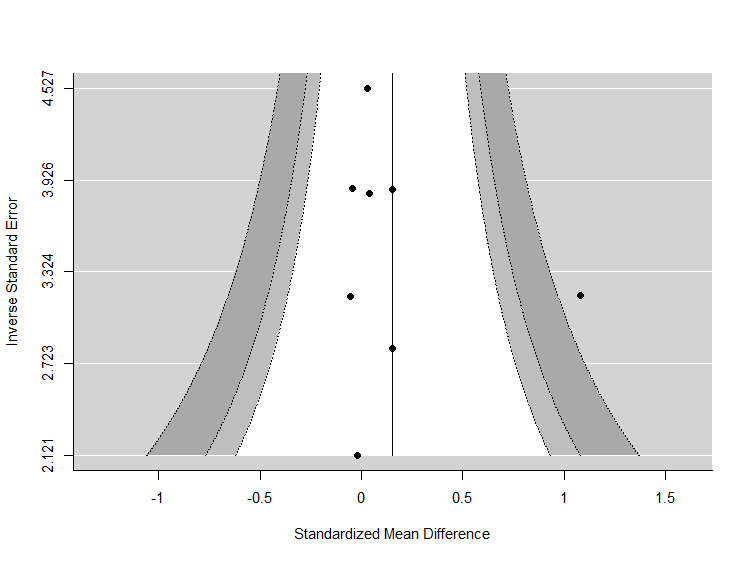** |
| --- | --- |
| **C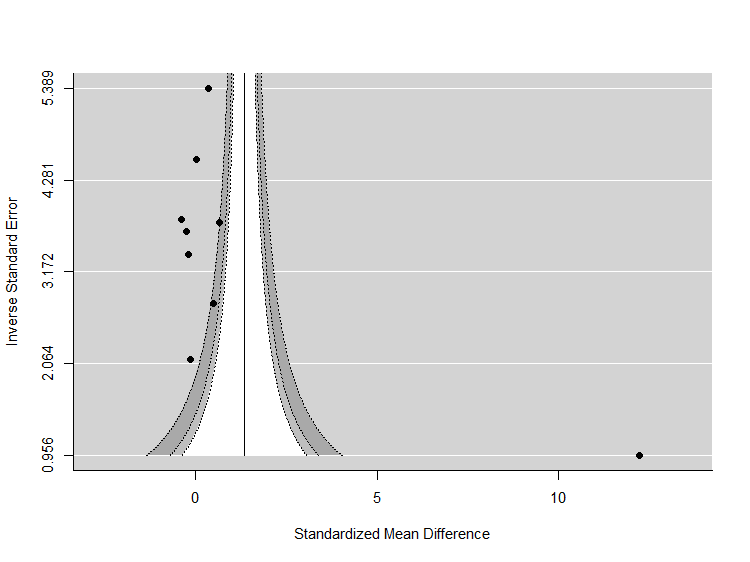** | **D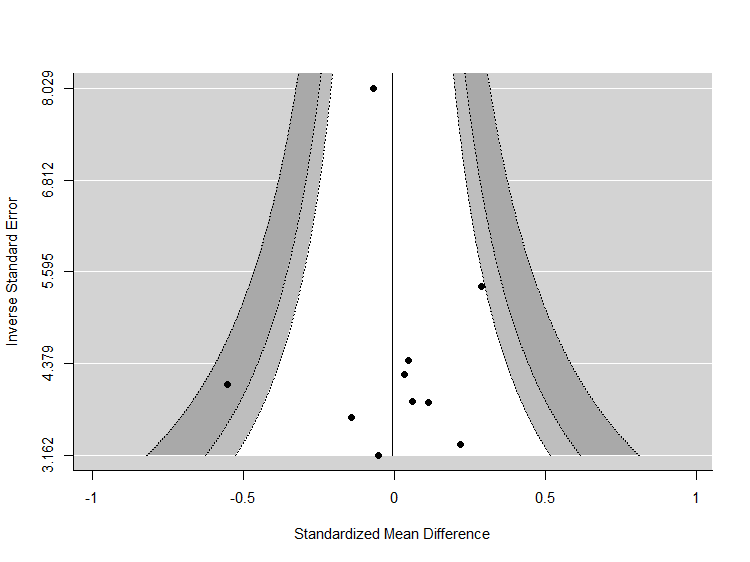** |

**Supplementary Figure 2.** Funnel plots for speed of attention publications (averaged repetitions) in **(A)** Simple RT, **(B)** Choice RT, **(C)** Digit Vigilance, **(D)** RVIP tasks.

| **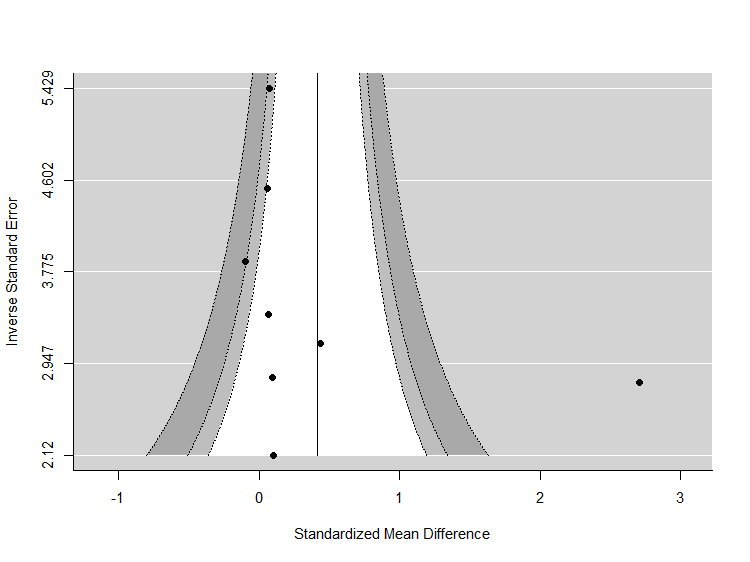A** | **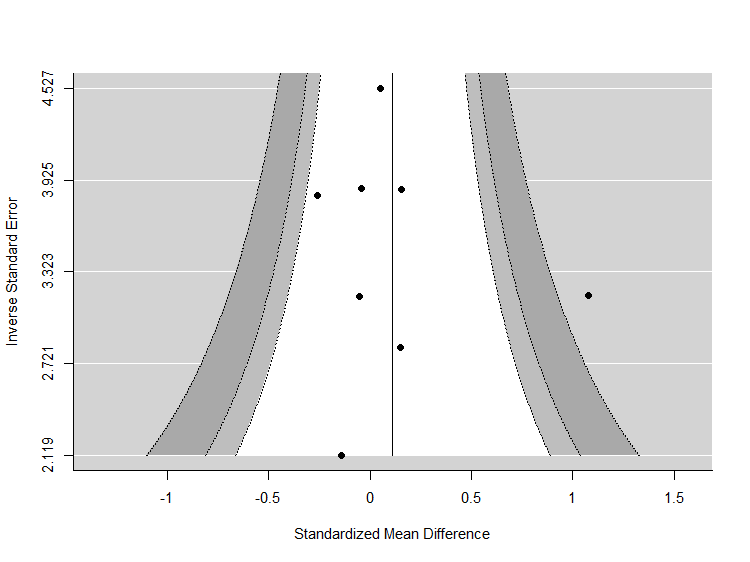B** |
| --- | --- |
| **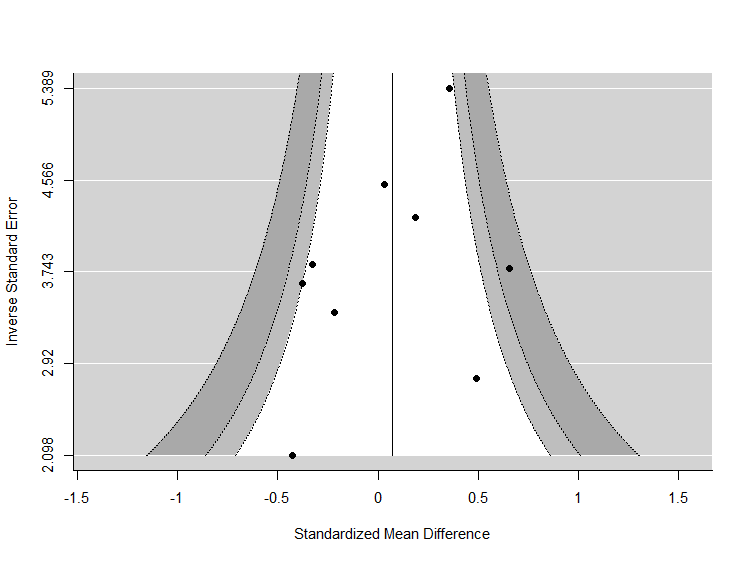C** | **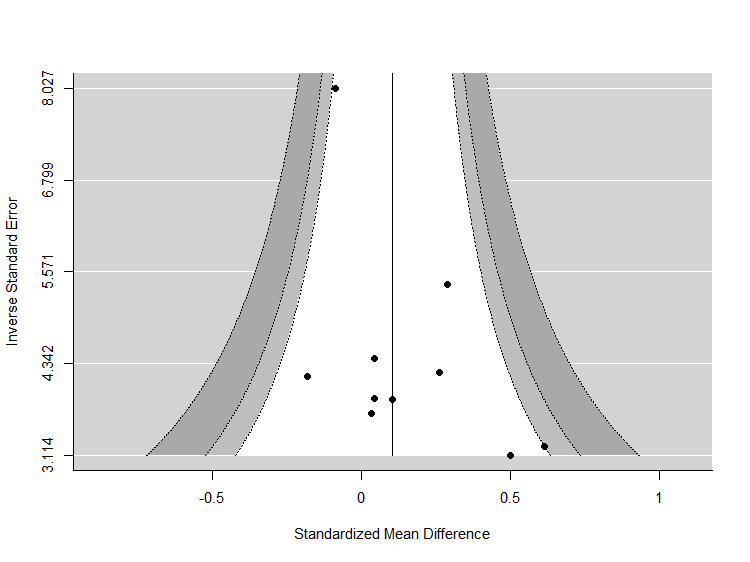D** |

**Supplementary Figure 3.** Funnel plots for speed of attention publications (last repetitions) in **(A)** Simple RT, **(B)** Choice RT, **(C)** Digit Vigilance, **(D)** RVIP tasks.

| **A**  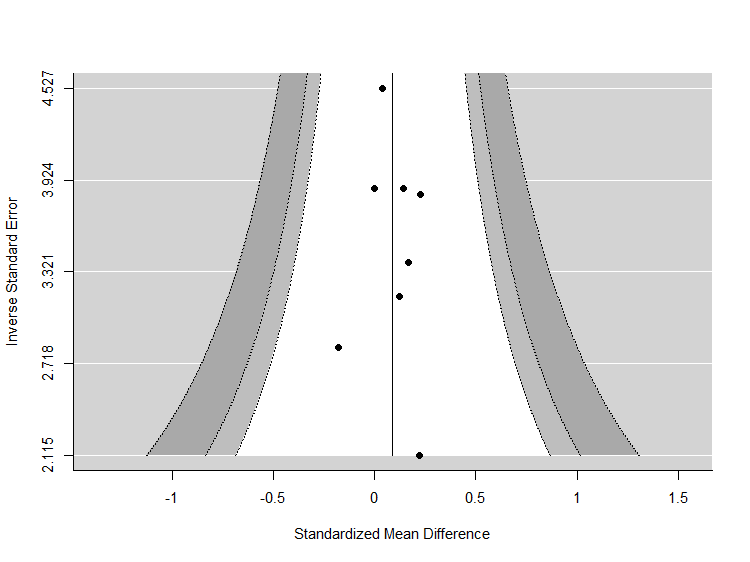 | |
| --- | --- |
| **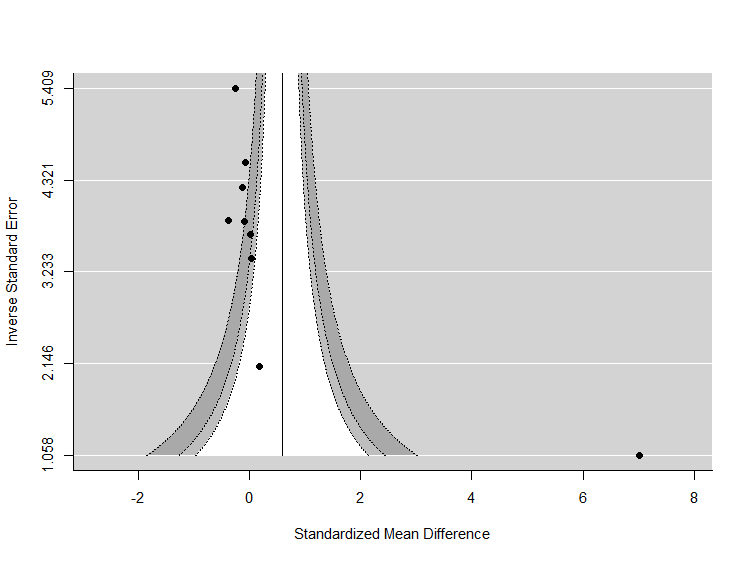B** | **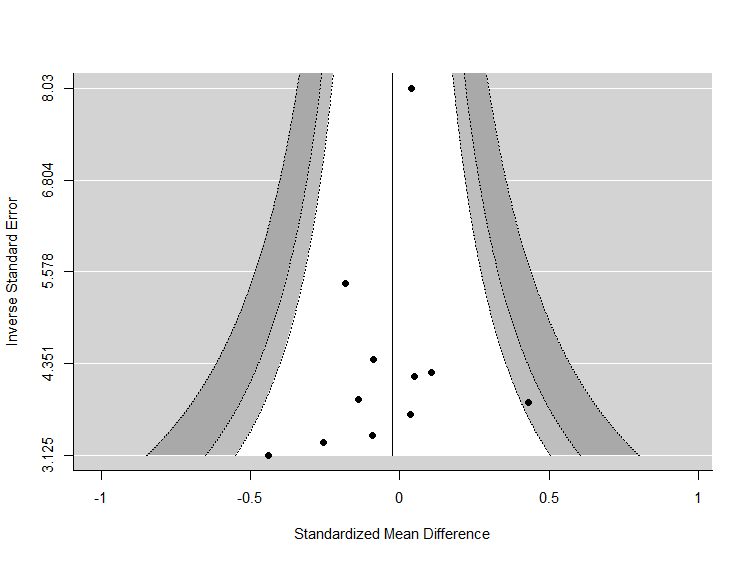C** |

**Supplementary Figure 4.** Funnel plots for accuracy of attention publications (averaged repetitions) in **(A)** Choice RT, **(B)** Digit Vigilance, **(C)** RVIP tasks.

| **A**  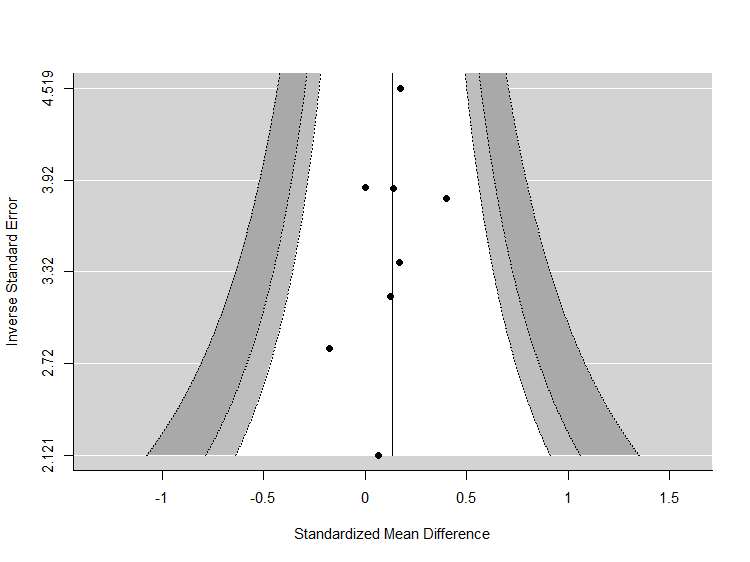 | |
| --- | --- |
| **B**  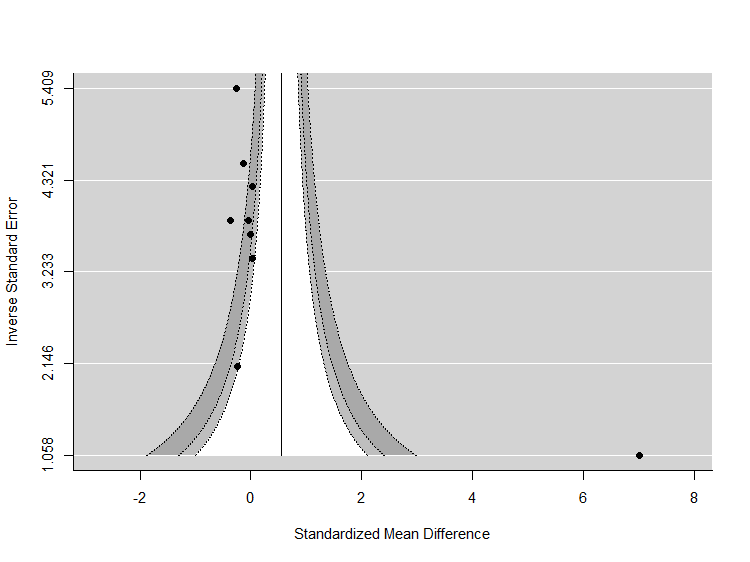 | **C**  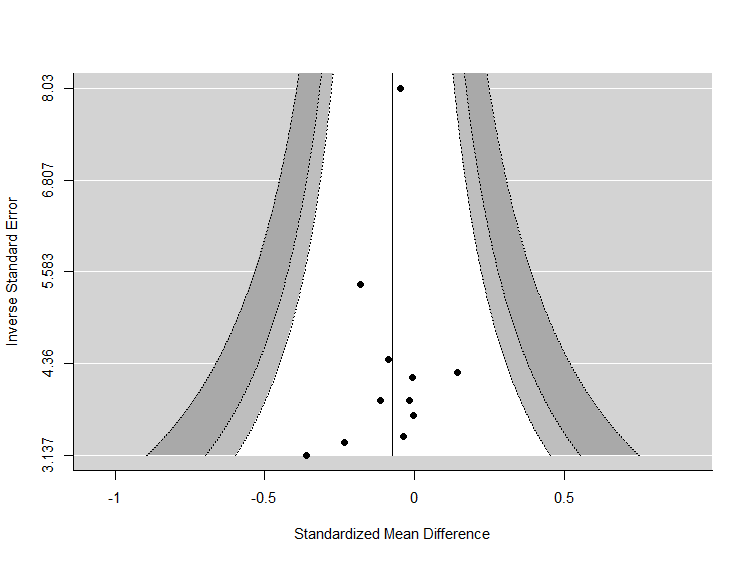 |

**Supplementary Figure 5.** Funnel plots for accuracy of attention publications (last repetitions) in **(A)** Choice RT, **(B)** Digit Vigilance, **(C)** RVIP tasks.
