# Supplementary material for: Acute Effects of Polyphenols on Human Attentional Processes: A Systematic Review and Meta-Analysis
Source: Front Neurosci. 2021 May 24;15:678769. doi: 10.3389/fnins.2021.678769 (PMC8180591; doi:10.3389/fnins.2021.678769)
Supplement: Supplementary file 2 [file Table_2.DOCX]

***Supplementary Results***

# **1. Effect of source of polyphenols – Repetitions averaged**

## **1.1. Speed following berry consumption: Meta-analyses could not be conducted for the majority of the tasks and for other polyphenol sources due to low number of studies (n≤ 2).**

### **1.1.1. Digit Vigilance**

**
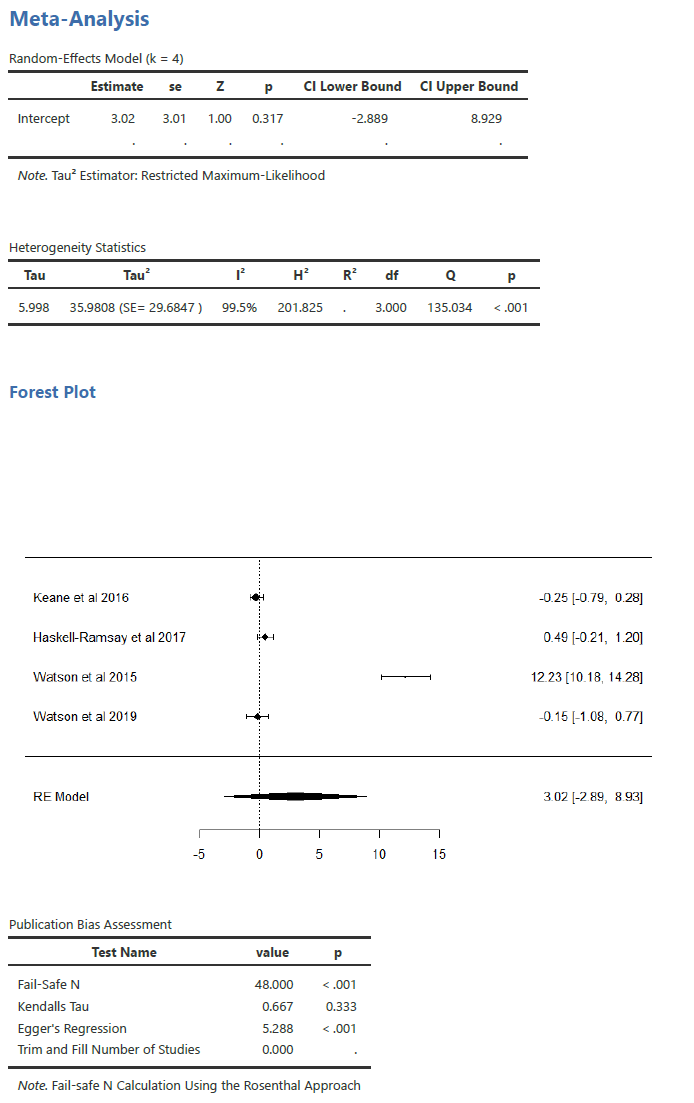
**

### **1.1.2. RVIP**

**
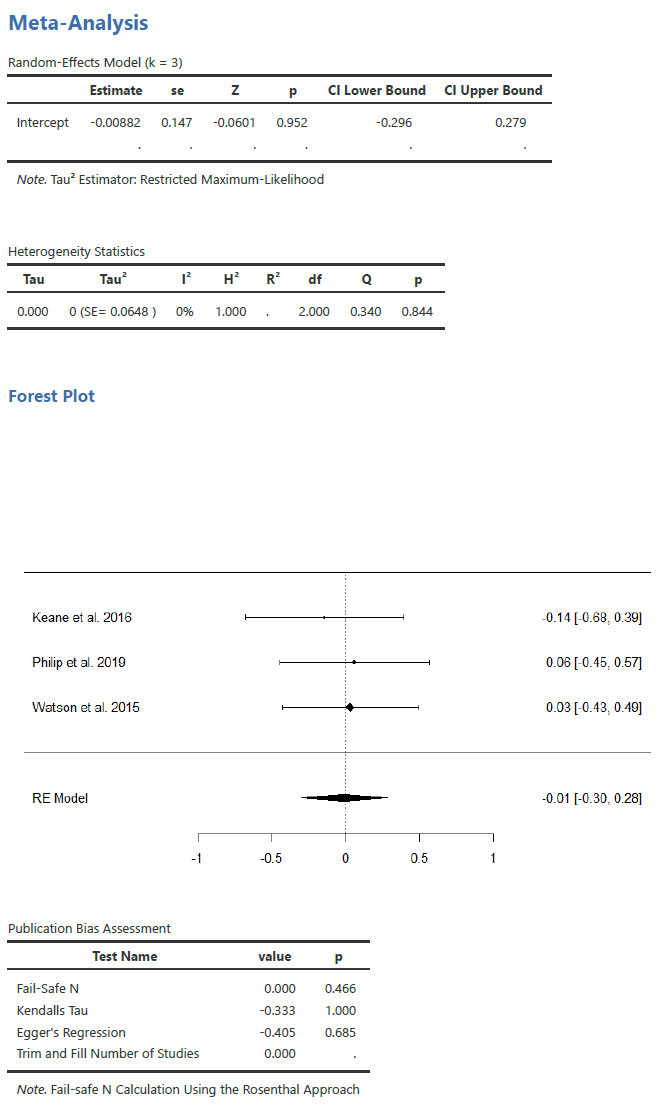
**

## **1.2. Accuracy following berry consumption: Meta-analyses could not be conducted for the majority of the tasks and for other polyphenol sources due to low number of studies (n≤ 2).**

### **1.2.1. Digit Vigilance**

**
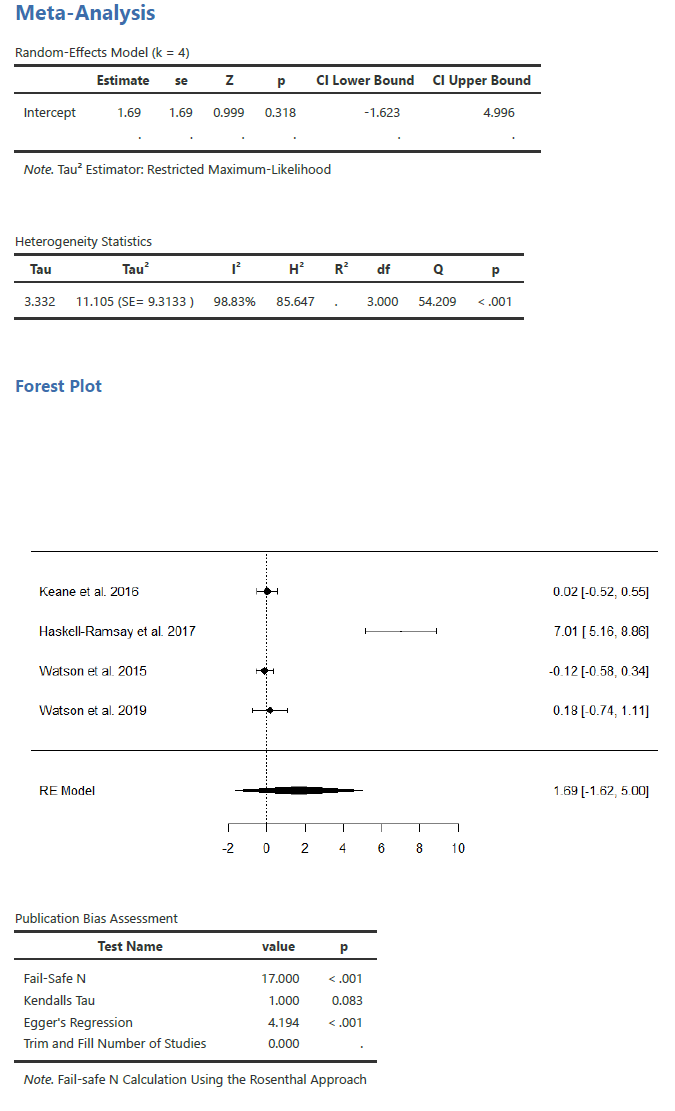
**

### **1.2.2. RVIP**

**
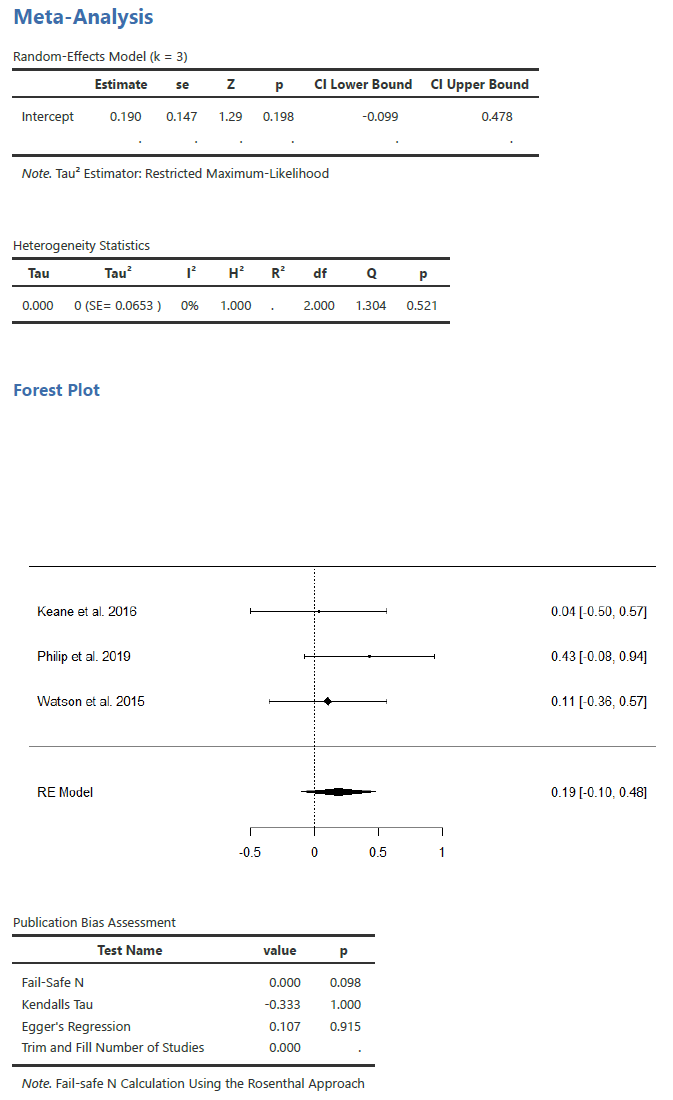
**

# **2. Effect of age – Repetitions averaged**

## **2.1. Speed in Young Participants**

### **2.1.1. Simple RT**

**
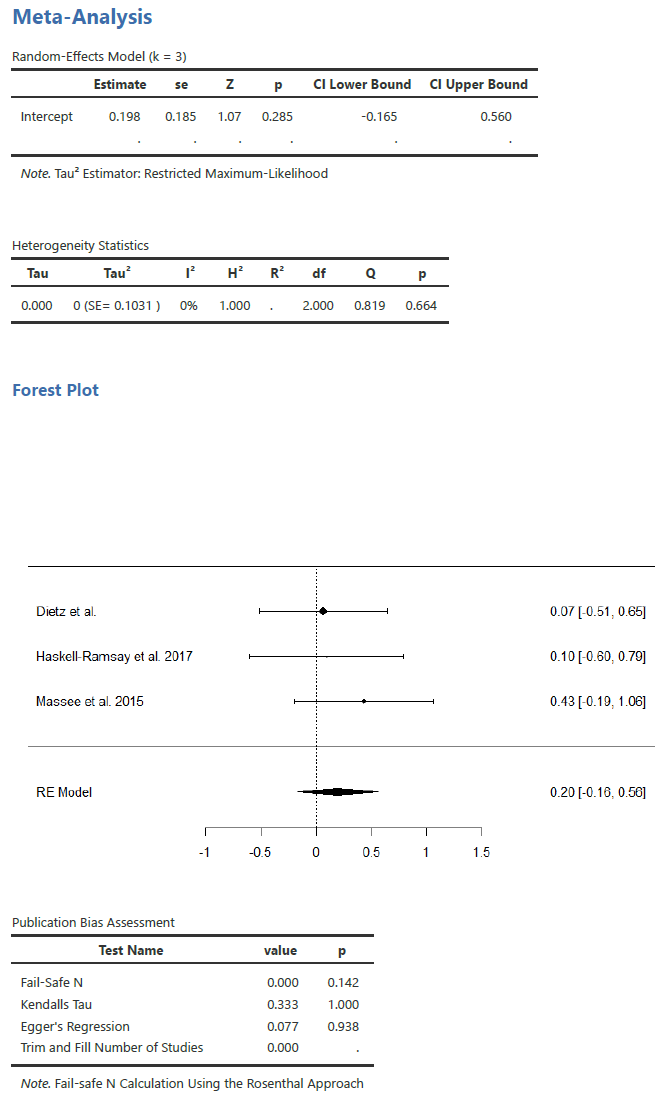
**

### **2.1.2. Choice RT**

**
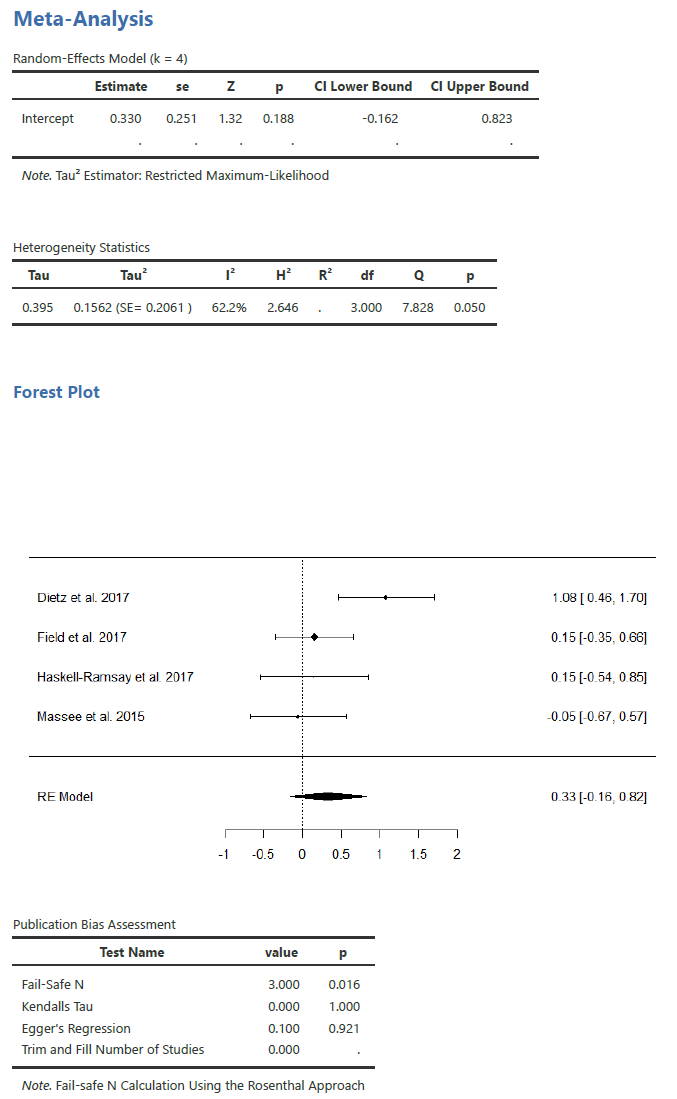
**

### **2.1.3. Digit Vigilance**

**
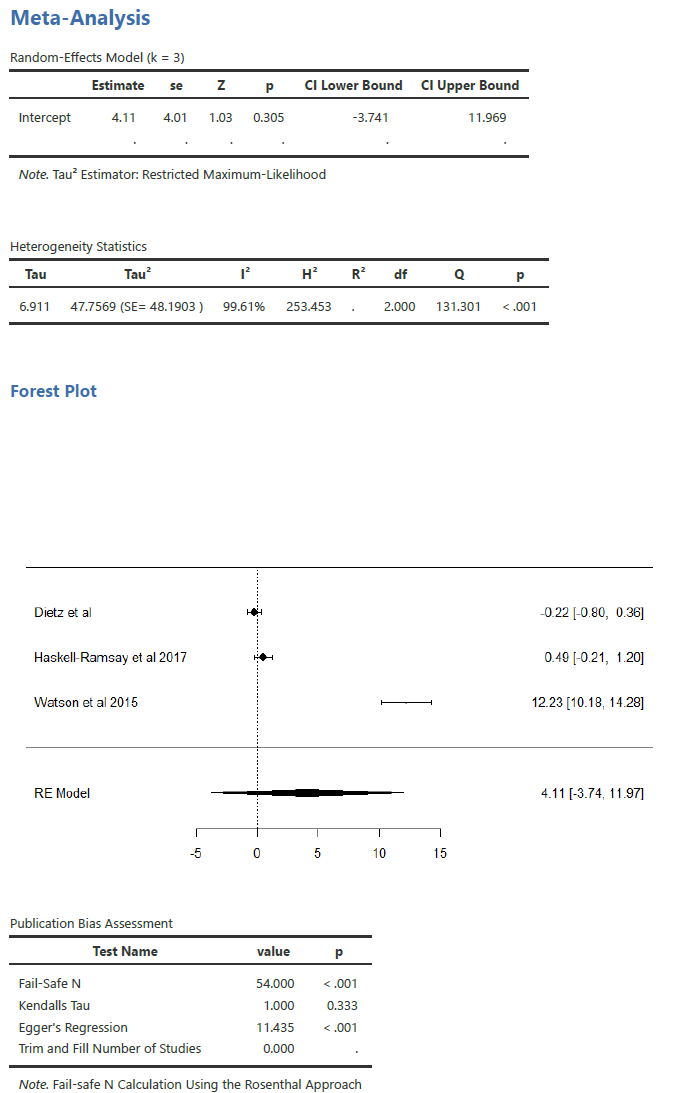
**

### **2.1.4. RVIP**

**
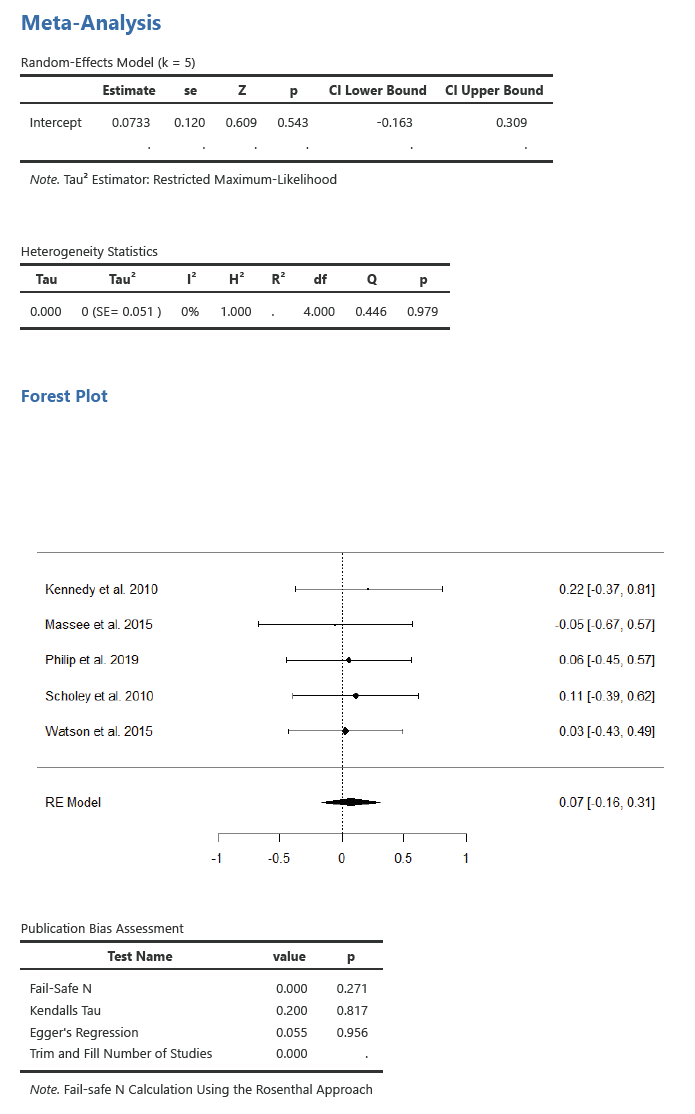
**

## **2.2. Accuracy in Young participants**

### **2.2.1. Choice RT**

**
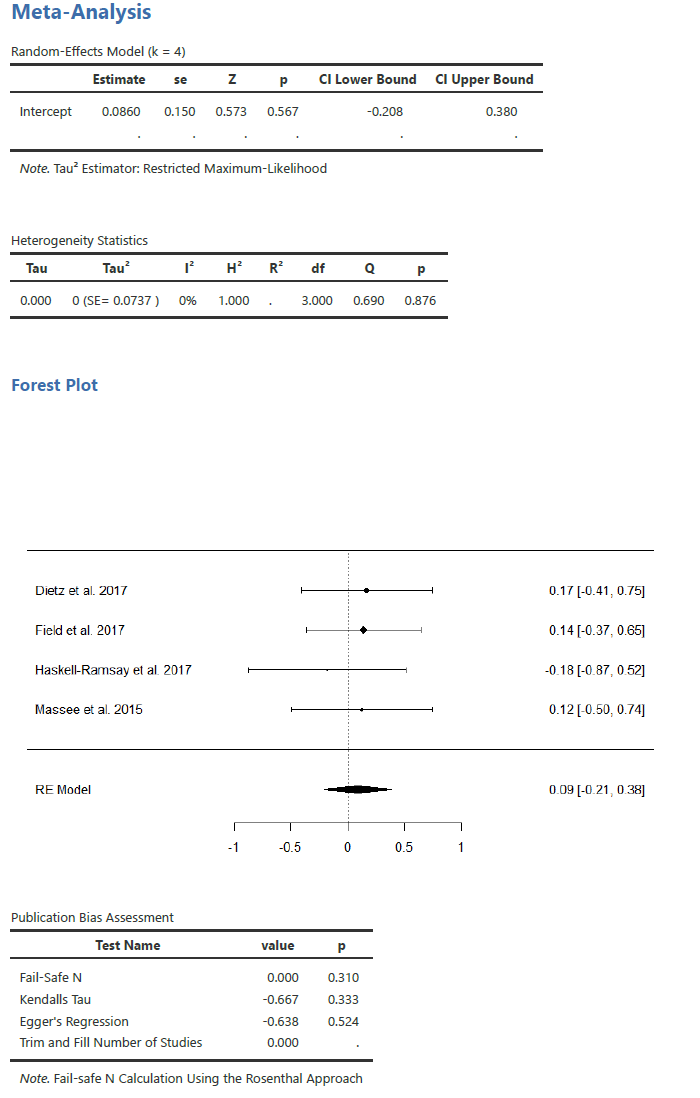
**

### **2.2.2. Digit Vigilance**

**
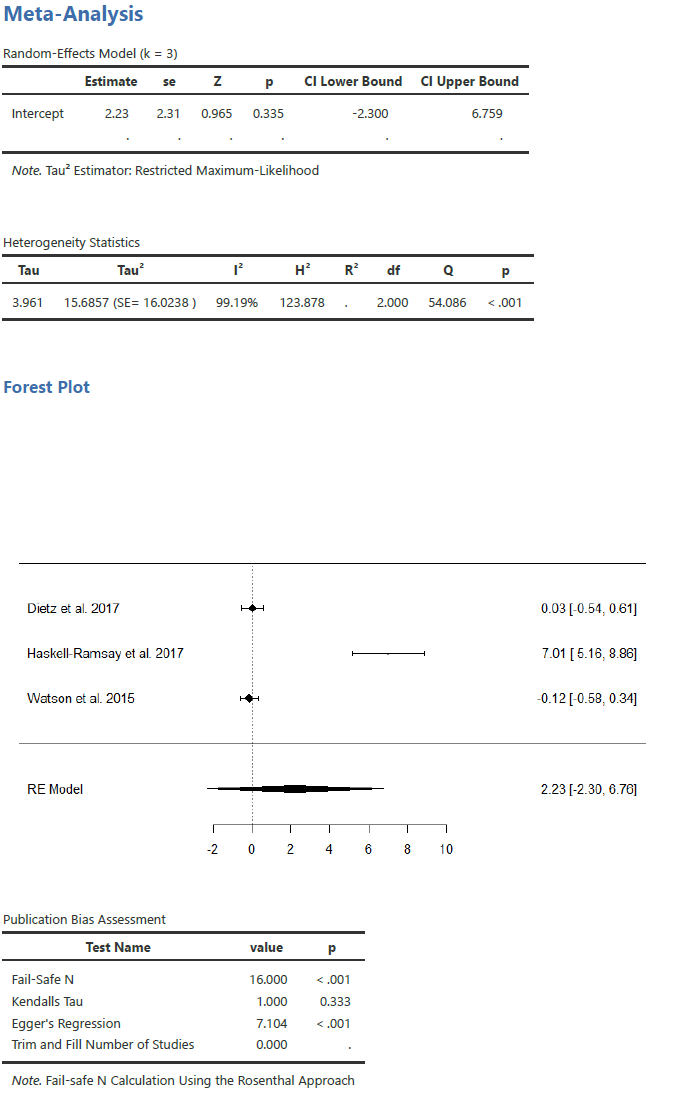
**

### **2.2.3. RVIP**

**
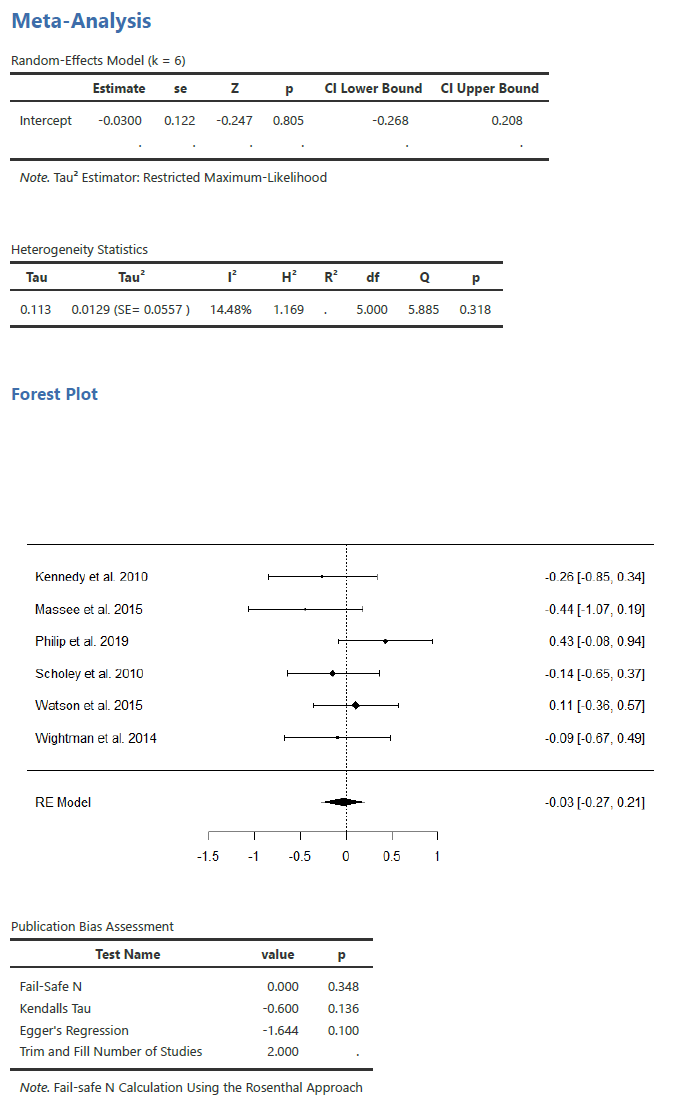
**

## **2.3. Speed in Old Participants: Meta-analyses could not be conducted for the majority of the tasks due to low number of studies (n≤ 2).**

### **2.3.1. RVIP**

**
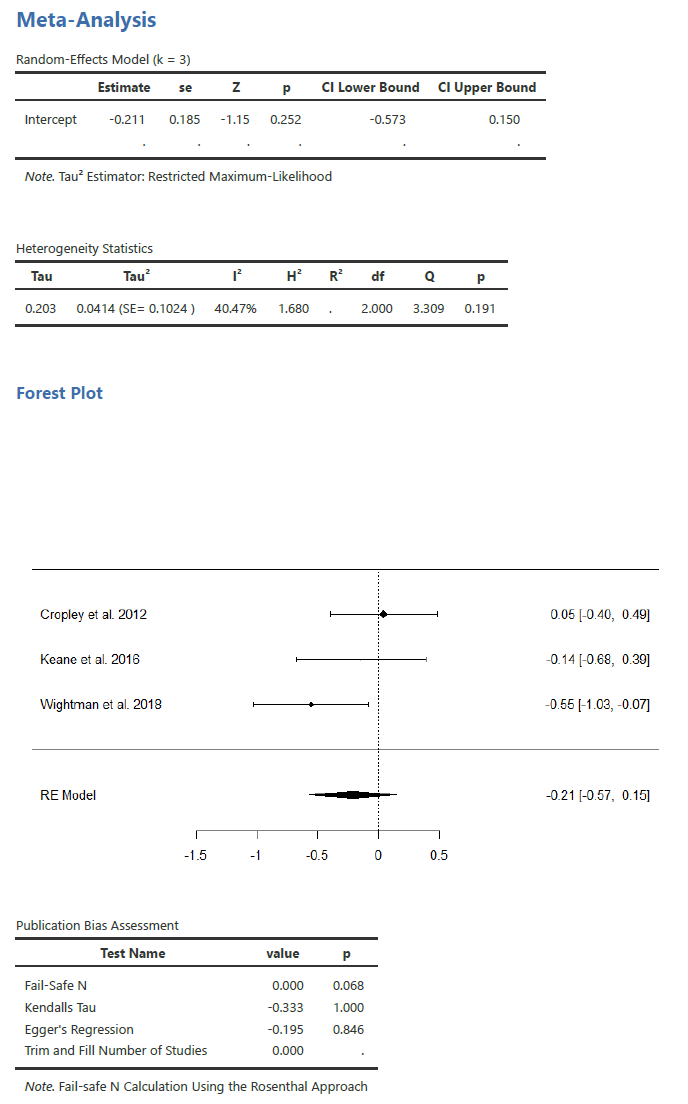
**

## **2.4. Accuracy in Old Participants: Meta-analyses could not be conducted for the majority of the tasks due to low number of studies (n≤ 2).**

### **2.4.1. RVIP**

**
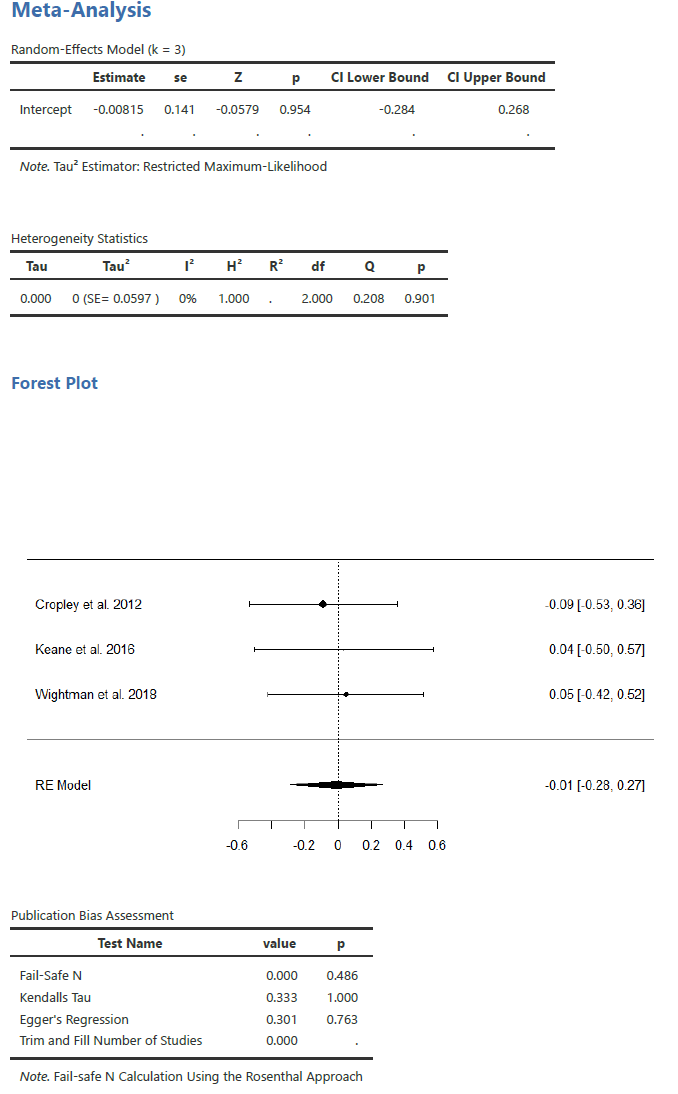
**

# **3. Effect of source of polyphenols – Last repetition only**

## **3.1. Speed following berry consumption: Meta-analyses could not be conducted for the majority of the tasks and for other polyphenols sources due to low number of studies (n≤ 2).**

### **3.1.1. Digit Vigilance**

**
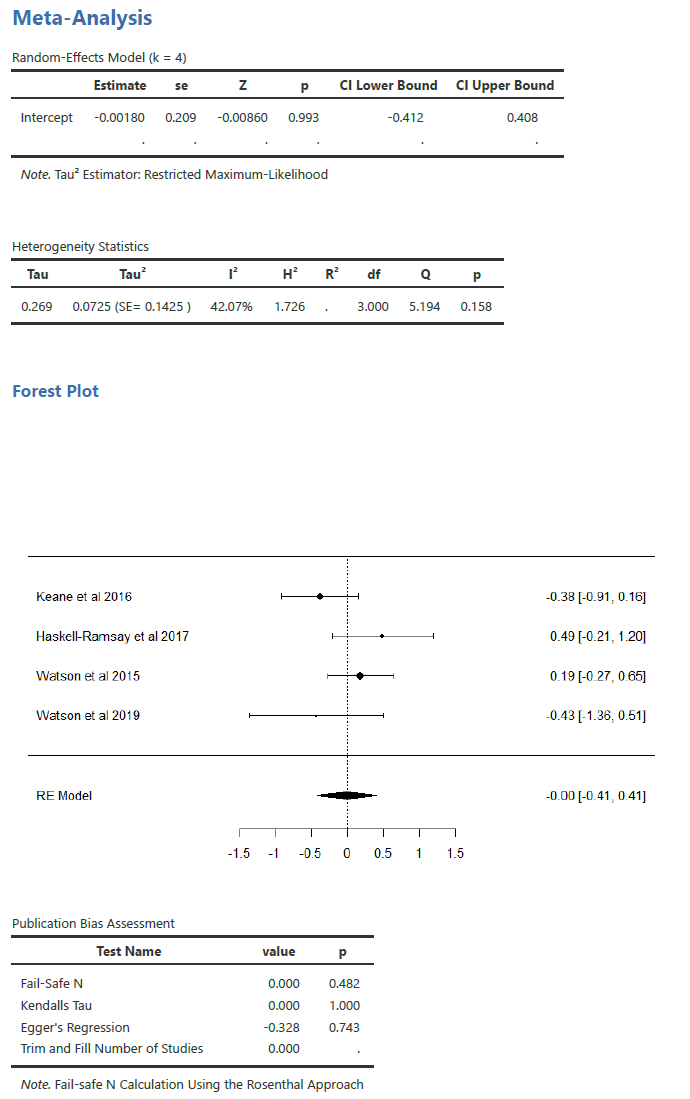
**

### **3.1.2. RVIP**

**
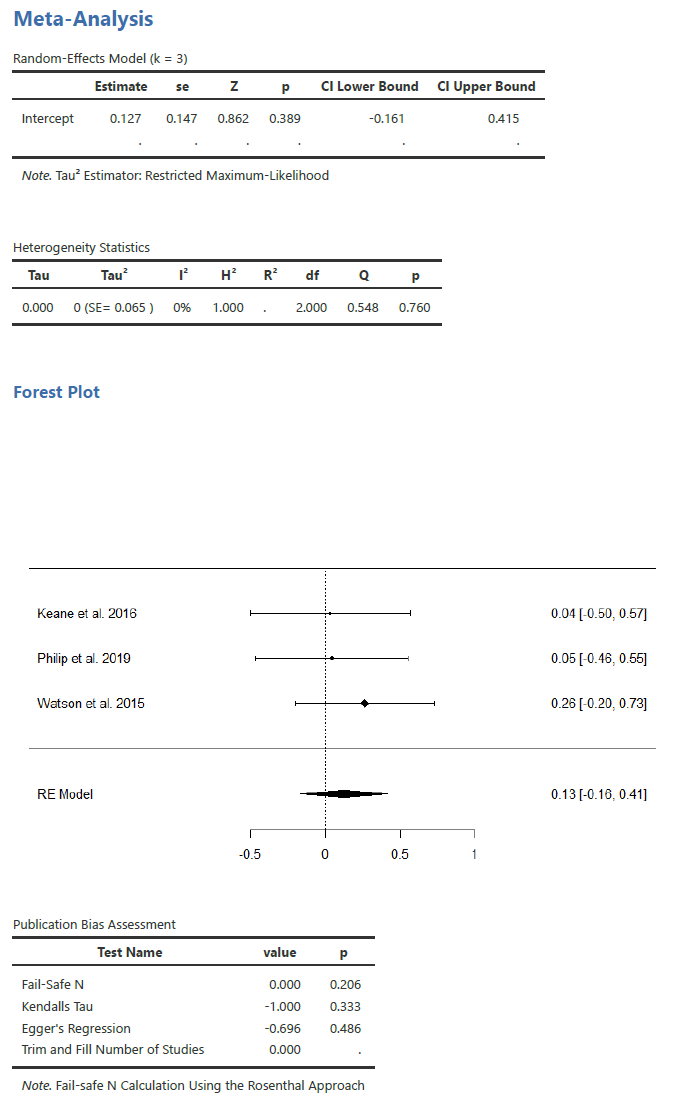
**

## **3.2. Accuracy following berry consumption: Meta-analyses could not be conducted for the majority of the tasks and for other polyphenols sources due to low number of studies (n≤ 2).**

### **3.2.1. Digit Vigilance**

**
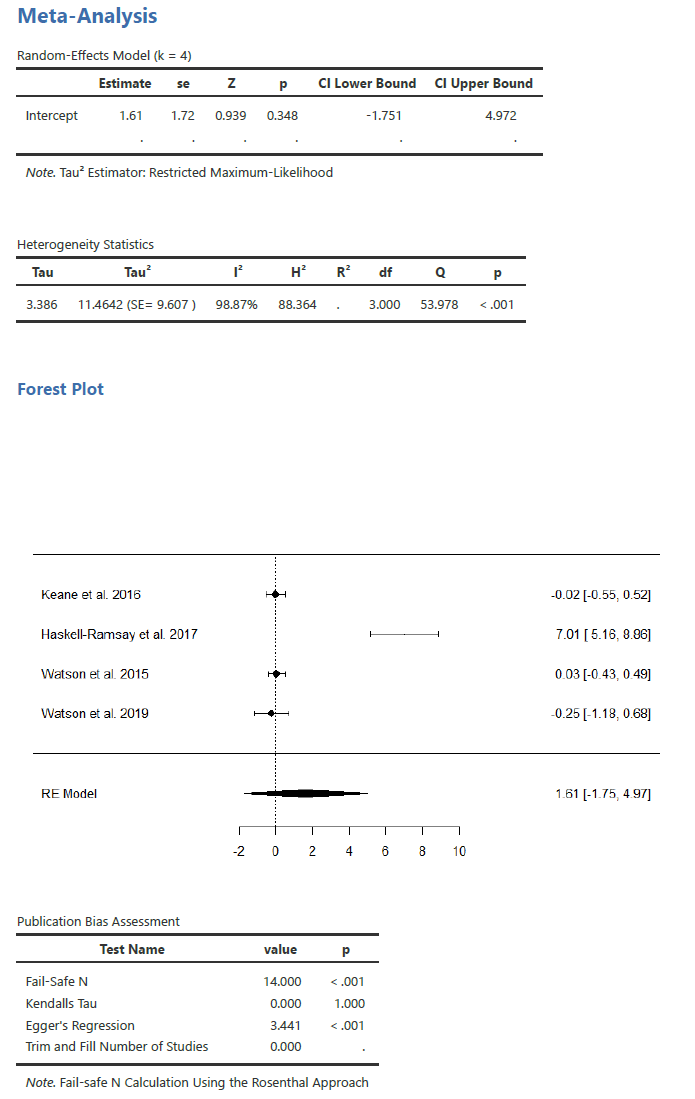
**

### **3.2.2. RVIP**

**
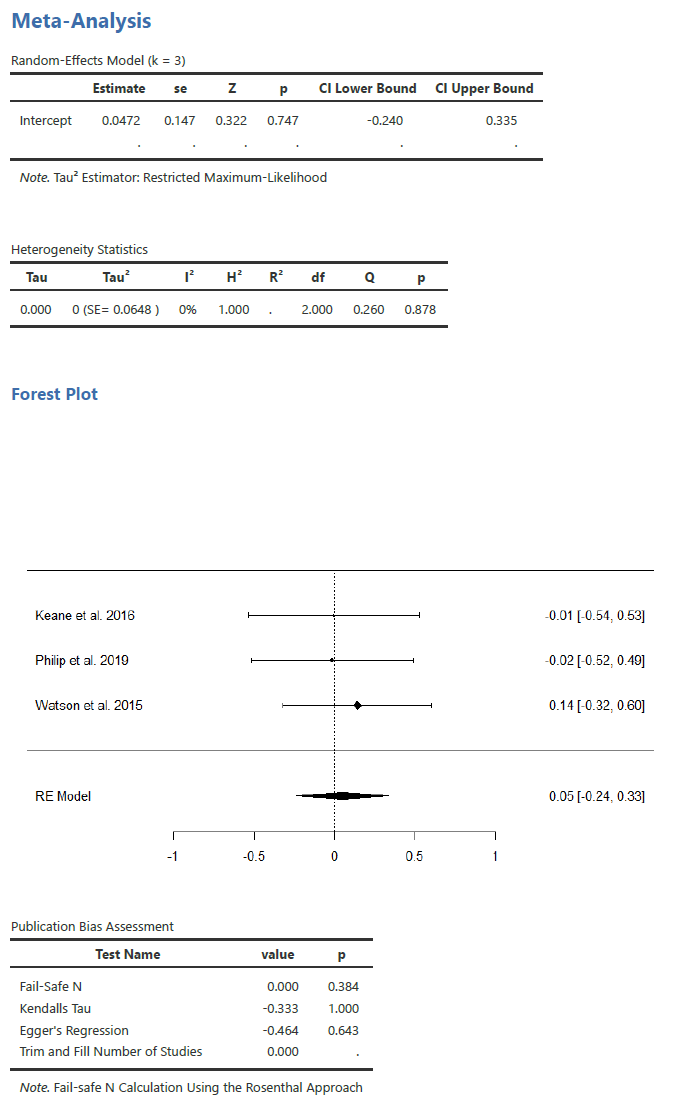
**

# **4. Effect of age – Last repetition only**

## **4.1. Speed in Young Participants**

### **4.1.1. Simple RT**

**
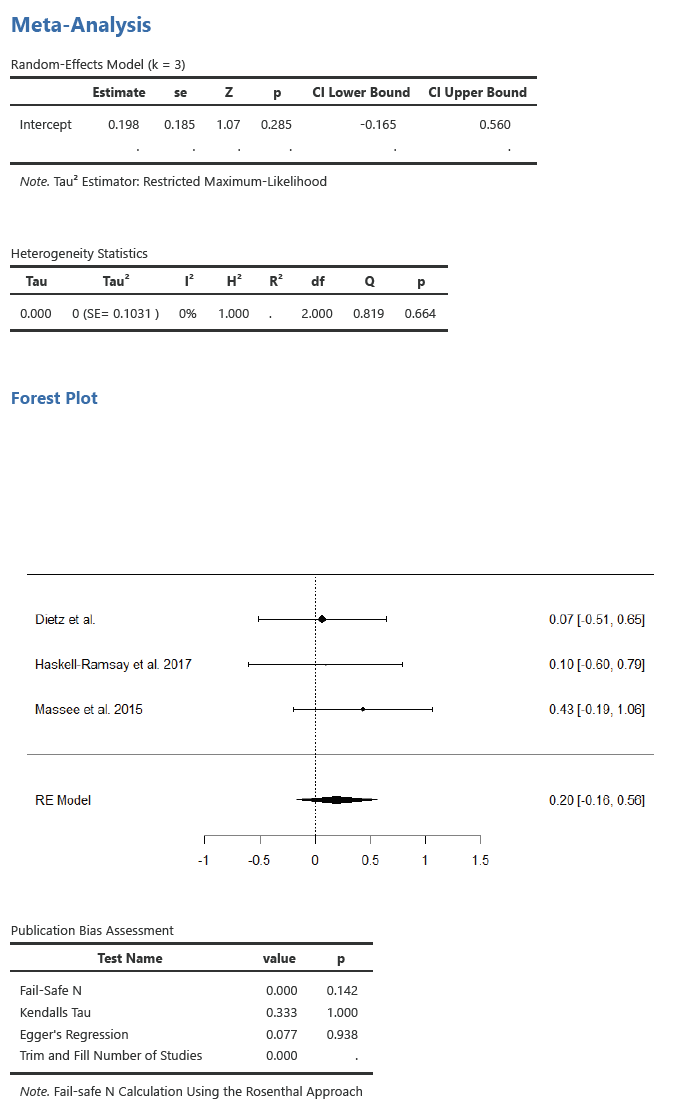
**

### **4.1.2. Choice RT**

**
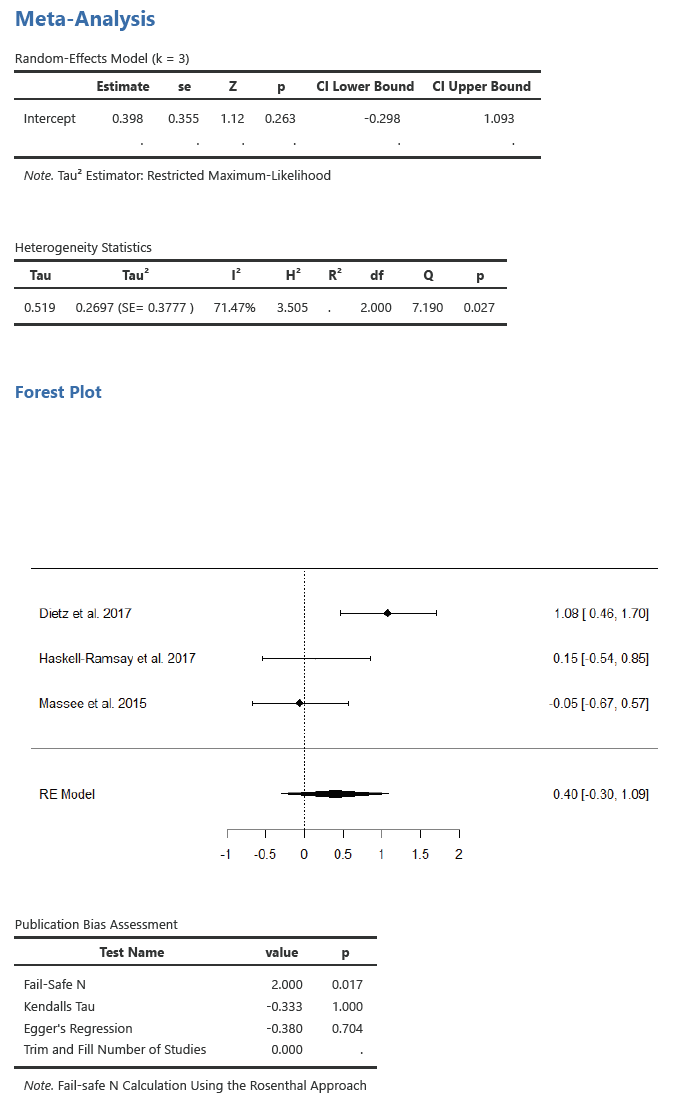
**

### **4.1.3. Digit Vigilance**

**
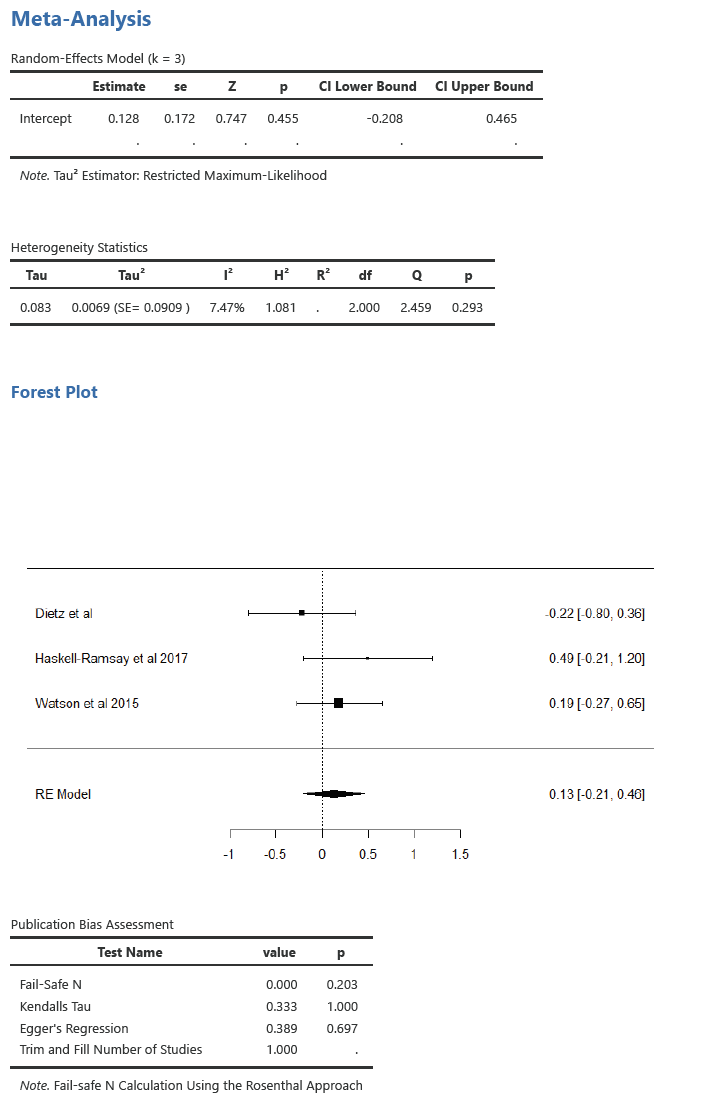
**

## **4.2. Accuracy in Young Participants**

### **4.2.1. Choice RT**

**
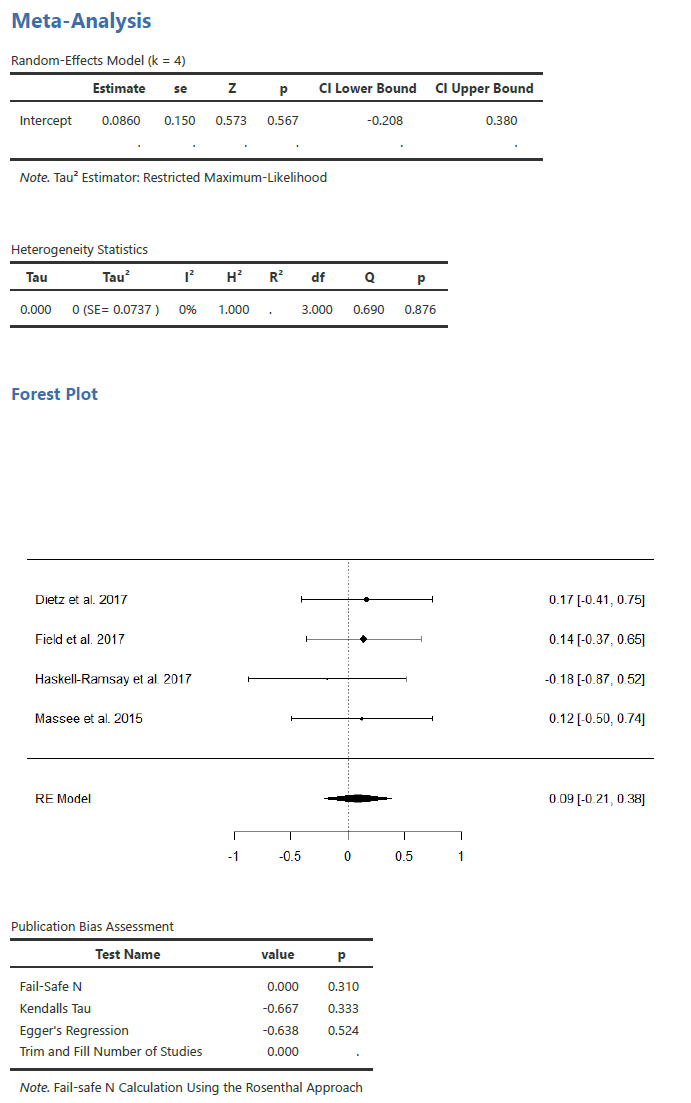
**

### **4.2.2. Digit Vigilance**

**
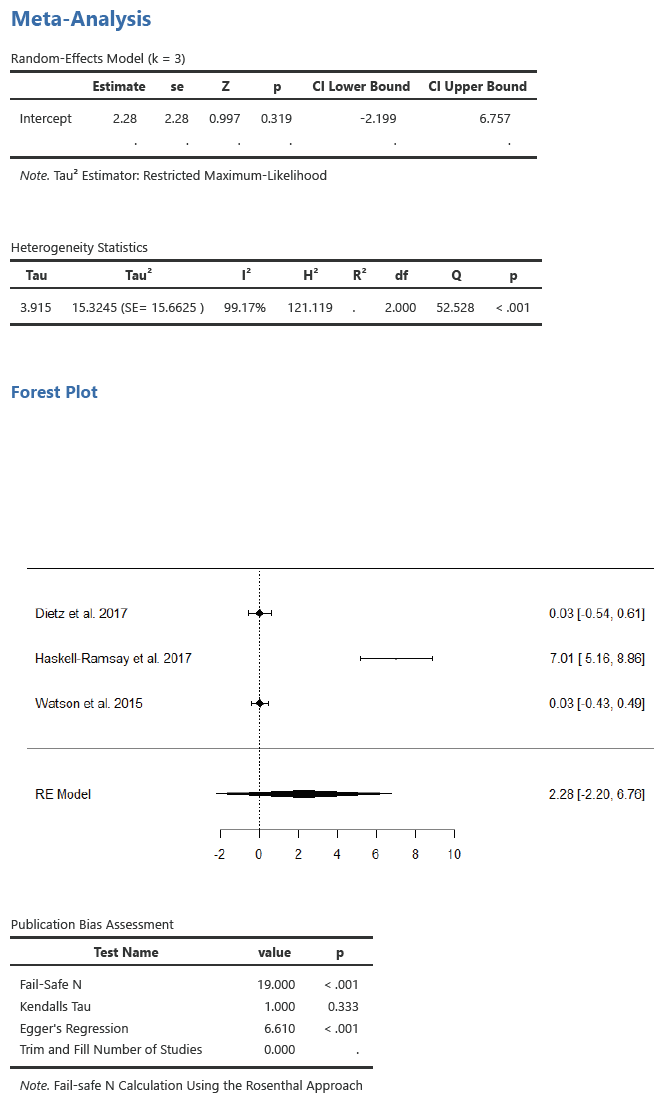
**

### **4.2.3. RVIP**

**
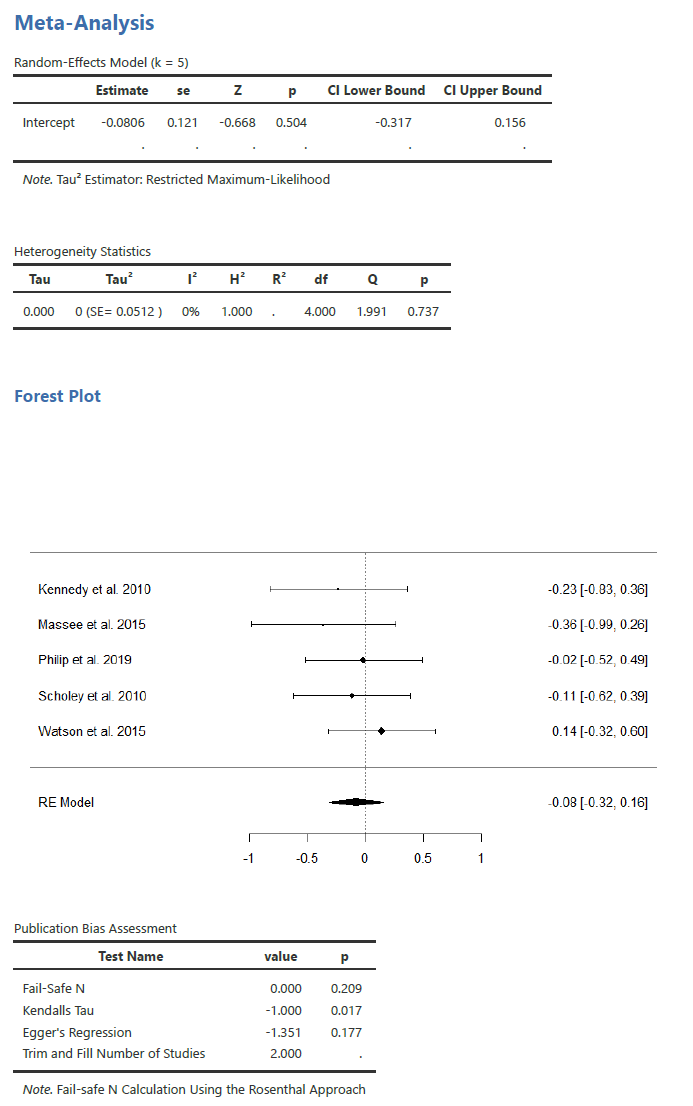
**

## **4.3. Speed in Old Participants: Meta-analyses could not be conducted for the majority of the tasks due to low number of studies (n≤ 2).**

### **4.3.1. RVIP**

**
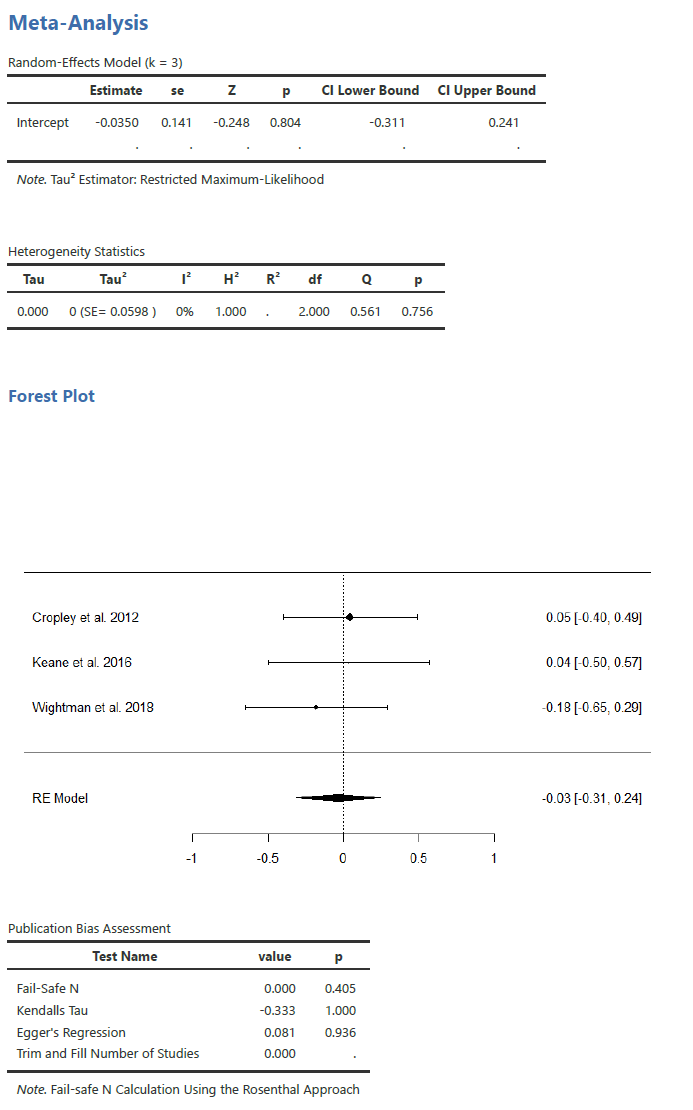
**

## **4.4. Accuracy in Old Participants: Meta-analyses could not be conducted for the majority of the tasks due to low number of studies (n≤ 2).**

### **4.4.1 RVIP**

**
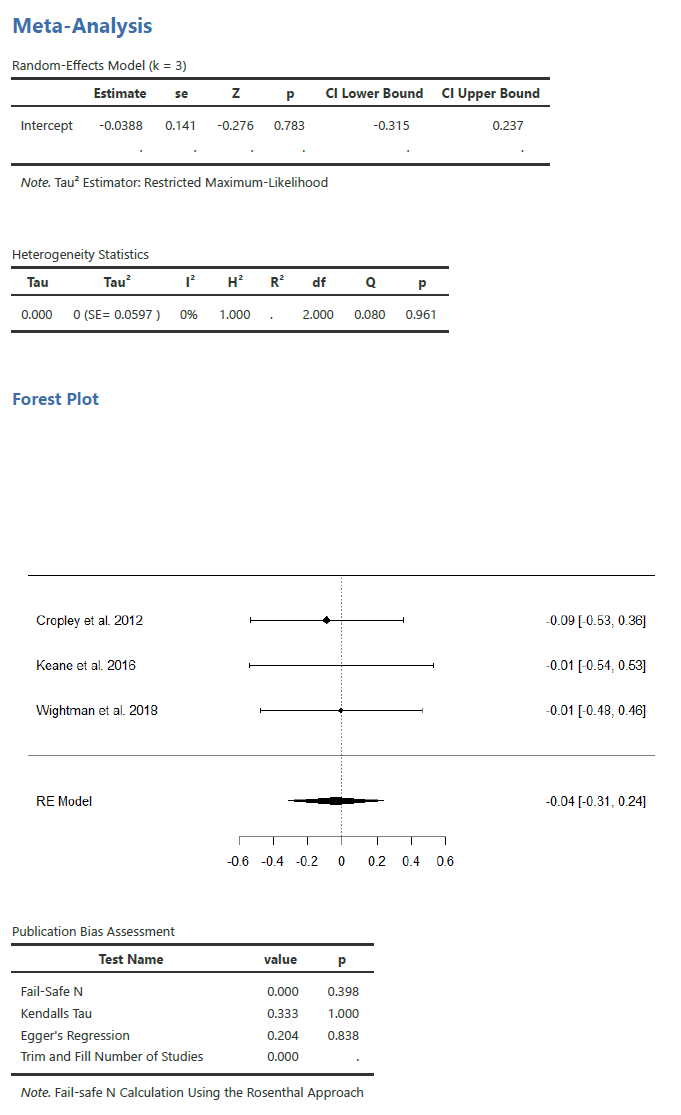
**
